# DnaK duplication and specialization in bacteria correlates with increased proteome complexity

**DOI:** 10.1128/msystems.01154-23

**Published:** 2024-03-26

**Authors:** Zhuo Pan, Li Zhuo, Tian-yu Wan, Rui-yun Chen, Yue-zhong Li

**Affiliations:** 1 State Key Laboratory of Microbial Technology, Institute of Microbial Technology, Shandong University, Qingdao, China; 2 Suzhou Research Institute, Shandong University, Suzhou, China; Chinese Academy of Sciences, Shanghai, China

**Keywords:** DnaK, duplication, *Myxococcus xanthus *DK1622, substrate spectrum, inter-swapping, prokaryotes, proteomic complexity

## Abstract

The chaperone 70 kDa heat shock protein (Hsp70) is important for cells from bacteria to humans to maintain proteostasis, and all eukaryotes and several prokaryotes encode Hsp70 paralogs. Although the mechanisms of Hsp70 function have been clearly illuminated, the function and evolution of Hsp70 paralogs is not well studied. DnaK is a highly conserved bacterial Hsp70 family. Here, we show that *dnaK* is present in 98.9% of bacterial genomes, and 6.4% of them possess two or more DnaK paralogs. We found that the duplication of *dnaK* is positively correlated with an increase in proteomic complexity (proteome size, number of domains). We identified the interactomes of the two DnaK paralogs of *Myxococcus xanthus* DK1622 (MxDnaKs), which revealed that they are mostly nonoverlapping, although both prefer α and β domain proteins. Consistent with the entire *M. xanthus* proteome, MxDnaK substrates have both significantly more multi-domain proteins and a higher isoelectric point than that of *Escherichia coli*, which encodes a single DnaK homolog. MxDnaK1 is transcriptionally upregulated in response to heat shock and prefers to bind cytosolic proteins, while MxDnaK2 is downregulated by heat shock and is more associated with membrane proteins. Using domain swapping, we show that the nucleotide-binding domain and the substrate-binding β domain are responsible for the significant differences in DnaK interactomes, and the nucleotide binding domain also determines the dimerization of MxDnaK2, but not MxDnaK1. Our work suggests that bacterial DnaK has been duplicated in order to deal with a more complex proteome, and that this allows evolution of distinct domains to deal with different subsets of target proteins.

**IMPORTANCE:**

All eukaryotic and ~40% of prokaryotic species encode multiple 70 kDa heat shock protein (Hsp70) homologs with similar but diversified functions. Here, we show that duplication of canonical Hsp70 (DnaK in prokaryotes) correlates with increasing proteomic complexity and evolution of particular regions of the protein. Using the *Myxococcus xanthus* DnaK duplicates as a case, we found that their substrate spectrums are mostly nonoverlapping, and are both consistent to that of *Escherichia coli* DnaK in structural and molecular characteristics, but show differential enrichment of membrane proteins. Domain/region swapping demonstrated that the nucleotide-binding domain and the β substrate-binding domain (SBDβ), but not the SBDα or disordered C-terminal tail region, are responsible for this functional divergence. This work provides the first direct evidence for regional evolution of DnaK paralogs.

## INTRODUCTION

The 70 kDa heat shock protein (Hsp70) is a widespread family of ATP-dependent molecular chaperones. Hsp70 homologs participate in a wide range of cellular processes by, in cooperation with other chaperones, folding *de novo* proteins or refolding/degrading misfolded proteins produced under heat or oxidative stress (1–3). A typical Hsp70 protein consists of three functional regions: an N-terminal 45 kDa nucleotide-binding domain (NBD), which is linked with a 15 kDa substrate binding domain (SBDβ), and then a 10 kDa α-helical lid domain (SBDα). Functionally, the NBD binds ATP, the SBDβ interacts with peptide substrates, and the SBDα functions as a lid to trap substrates in the polypeptide-binding cavity. In addition, some Hsp70 homologs have a highly disordered C-terminal tail (CTT), which directly follows the lid domain with an unclear function (4, 5). Two types of cochaperones cooperate with Hsp70 to allow its function: a J domain protein (JDP, i.e., DnaJ), which stimulates Hsp70 ATPase to assist substrate folding (6), and a nucleotide exchange factor (NEF), which promotes ADP dissociation and the release of folded substrates (7). The JDP binds to Hsp70 at the interface between the NBD and the SBDβ via the J domain, while the NEF binds to the NBD region (8, 9). After releasing the folded proteins and ADP, Hsp70 binds new client proteins and ATP to restart the allosteric cycle (10).

Based on conserved domains, Hsp70 proteins are divided into two superfamilies (11). The conserved domain cd10170 covers the NBD domain only, while the cl35085 superfamily domain stretches over both the NBD and SBD regions. Within the cl35085 superfamily, some proteins are highly conserved and cluster to form the PRK00290 family. These proteins are usually characterized as the canonical Hsp70 chaperone (DnaK proteins in bacteria). All eukaryotes and ~40% prokaryotes^-^encode multiple Hsp70 copies (8, 11). Hsp70 paralogs in the same cell usually play divergent cellular functions, but with an overlap to different extents depending on their similarity (12–14). For example, human HSC70 and HSP70 are 85% identical, and the phenotypes of their knockout mutants are different, which is consistent with their largely nonoverlapping substrates and highly distinct expression patterns (15). Ssb1 and Ssb2 are two Hsp70 homologs of *Saccharomyces cerevisiae*, sharing an almost identical sequence and, thus, an almost identical nascent substrate pool (16); the deletion of *Ssb1* or *Ssb2* is phenotypically silent, but double deletion of the two genes causes slow growth and sensitivity against cold (17). Hsp70 is also duplicated and plays distinct functions in many bacteria (8, 11). The functional diversification of distinct Hsp70 isoforms is suggested to be due to differential expression, abundance, subcellular localization, as well as substrate specificity (8), which has not been as extensively investigated. To date, in addition to the above examples in human and yeast, only two other organisms (*Escherichia coli* and *Mycobacterium smegmatis*) have experimentally determined DnaK clients, which are significantly different from each other not only in substrate number but also in substrate composition (18, 19).

*Myxococcus xanthus* DK1622 is the model strain of myxobacteria. This Gram-negative bacterium possesses a large set of paralogous genes for complex social behavior and environmental adaptation (20, 21). In our previous studies, we found *M. xanthus* strain DK1622 encodes 15 Hsp70 homologs, of which six genes encode cd10170 family proteins and nine genes encode cl35085 family proteins (including two PRK00290 proteins) (11). The two PRK00290 proteins (MXAN_3192 and MXAN_6671) are phylogenetically located in the same subbranch with the typical DnaK proteins of other bacteria like *E. coli*, *Bacillus subtilis,* and *Lactococcus lactis*, while the other cl35085 proteins and the cd10170 proteins are located on different branches from these typical DnaK homologs. Either of the two PRK00290 proteins, but not other *Myxococcus* Hsp70 homologs, can compensate for the functions of EcDnaK (DnaK of *E. coli*) in growth (11). MXAN_3192 (MxDnaK1), the only *hsp70* gene in DK1622 that is essential for viability, is highly expressed and induced by heat shock. In comparison, the transcription of MXAN_6671 (MxDnaK2) is low and decreases in response to heat shock. MxDnaK2 is non-essential, but its deletion increases sensitivity to oxidative stress and affects some social behaviors (11, 22, 23). While these two *Myxococcus* DnaK homologs appear to be functionally divergent, the mechanisms underlying this difference remain elusive.

In this study, we surveyed the distribution of DnaK homologs in prokaryotes and found that the presence of DnaK chaperone paralogs in bacteria is correlated with an increase in proteomic complexity. We identified the interactomes of the two DnaKs in *M. xanthus* DK1622 and compared the two interactomes with each other and with that of EcDnaK. We also performed region inter-swapping between MxDnaKs and analyzed the effect on their interactomes and cellular phenotypes. Although the substrate spectrums of the two DnaK paralogs possess highly similar SCOPs (Structural Classification of Proteins) folds, they recognize largely nonoverlapping substrates, with MxDnaK2 preferring membrane proteins. Finally, we determined that the functional specificity of DnaK paralogs is mainly driven by regional evolution of the NBD and SBDβ.

## RESULTS

### Presence of DnaK paralogs correlates with increasing proteomic complexity in prokaryotes

**Fig 1:**
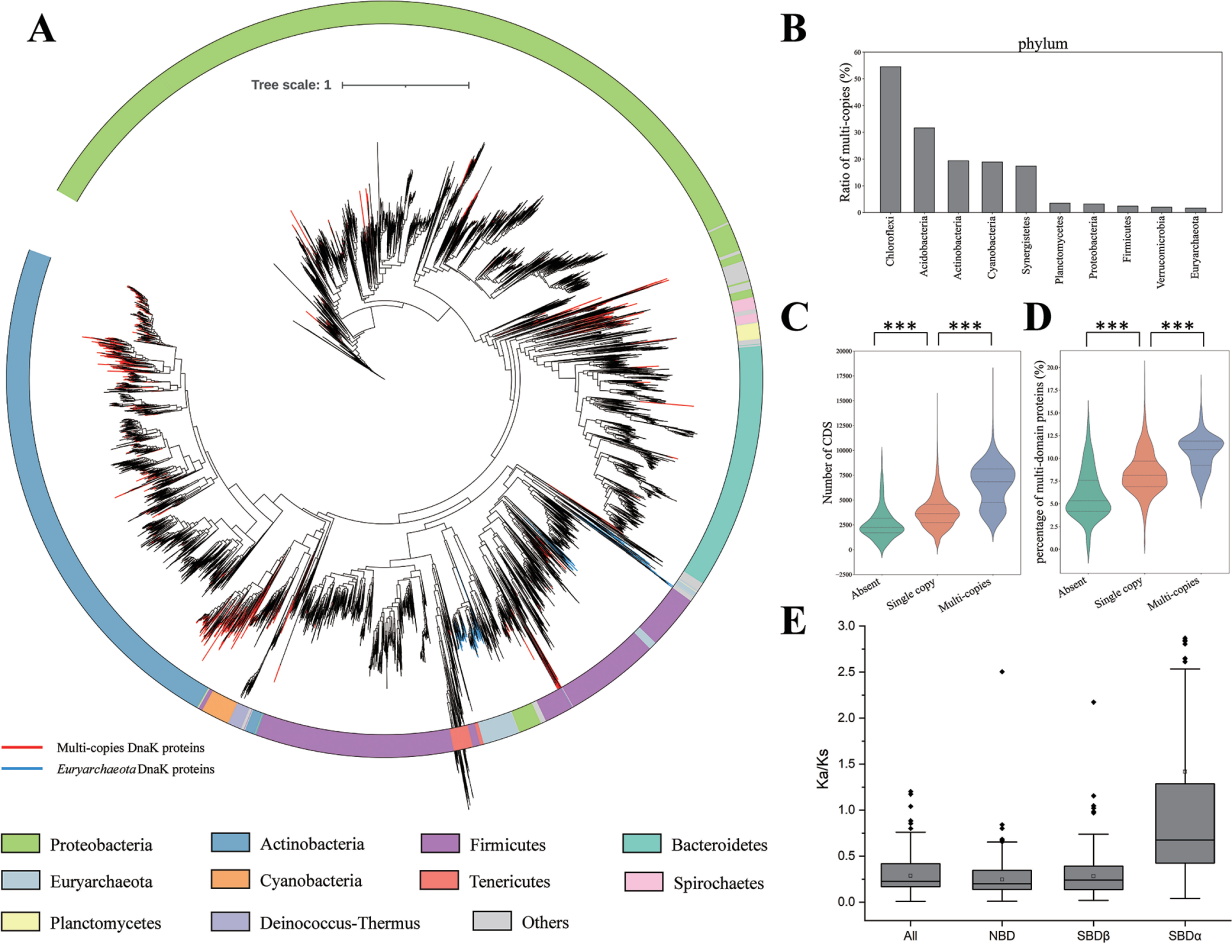
The distribution and characteristic analyses of DnaK from bacteria and archaea. (**A**) Phylogenetic analysis of 16,947 DnaK proteins from representative prokaryotes based on the neighbor-joining method by using the whole sequence of DnaK proteins. Multiple copies of DnaK in a single genome are marked with red branches, and the *Euryarchaeota* DnaK homologs are marked with blue branches. The top 10 phylum resources of DnaK are colored in the outer ring, while the others are in gray. (**B**) The top 10 phyla with high ratios of multiple *dnaK* copies in prokaryotes. (**C–D**) The coding sequence (CDS) number (**C**) and the proportion of multi-domain proteins (**D**) of genomes with zero, single copy, and multiple copies of *dnaK*. The *P*-value is based on the Mann-Whitney U-test. ***P < 0.001. (**E**) The Ka/Ks values for whole protein sequences, as well as the NBD, SBDβ, and SBDα regions of all *dnaK* pairs.

We screened for proteins with the PRK00290 domain in the genomes of representative prokaryotes and obtained a total of 16,947 putative DnaK homologs: 16,540 from 15,554 bacterial genomes and 407 from 529 archaeal genomes (Data set S1). The number of sequenced genomes varied greatly in different taxa. For example, the phyla of *Proteobacteria*, *Actinobacteria*, *Firmicutes,* and *Bacteroidetes* occupied 89.4% of the 16,083 prokaryotic genomes. Distribution of the DnaK homologs also greatly varied (Table S1). We found that 98.9% of the bacterial genomes contained at least one *dnaK* gene, while *dnaK* was present in 75.6% of the archaeal genomes. Many archaeal taxa lacked *dnaK*. For example, there was no *dnaK* gene in the 58 *Crenarchaeota* genomes, but the *Euryarchaeota* genomes possessed zero, single, or multiple *dnaK* genes. Figure 1A shows a phylogenetic tree of the 16,947 identified DnaK proteins. The DnaK phylogeny was mostly in accordance with the phylogeny of prokaryotic organisms, but with some exceptions. For example, the *Cyanobacteria* DnaK clade contained some DnaK branches with *Firmicutes* homologs. The *Euryarchaeota* DnaK proteins were divided into three groups (blue branches in Fig. 1A), which were mixed with the *Firmicutes* homologs, suggesting a possible evolutionary relationship.

There were 999 representative bacterial genomes (6.4% of the total) possessing two or more *dnaK* genes, and seven archaea encoding multiple DnaK proteins. Phylogenetic analysis showed that the distribution of DnaK paralogs was considerably wide but uneven in prokaryotes (red branches in Fig. 1A). At the phylum level, five bacterial phyla had a high rate of strains with multiple copies of the *dnaK* gene (phyla with less than 10 genomes were excluded from this analysis). *Chloroflexi* had the highest rate of *dnaK* paralogs (54.5% of 55 genomes), followed by *Acidobacteria* (31.6% of 38), *Actinobacteria* (19.4% in 3247), *Cyanobacteria* (18.9% in 180), and *Synergistetes* (17.4% of 23) (Fig. 1B). The other phyla had a low rate (<3.5%) or none with two *dnaK* genes (Table S1). The rate of possessing two copies of *dnaK* in some lower taxonomic units of bacteria was greatly increased, even reaching 100% (Fig. S1). For example, at the class level, the *Ktedonobacteria* and *Chloroflexia* of the *Chloroflexi* phylum had 88.2% and 72.7%, respectively, of strains with multiple copies of *dnaK*. At the order level, two copies of *dnaK* were detected in all the sequenced genomes of *Chloroflexales* (an order of *Chloroflexia*) or *Syntrophobacterales* of the *Deltaproteobacteria*.

We found that the number of coding sequences (CDSs) was significantly different in the genomes with zero, single, or multiple *dnaK* genes (Fig. 1C). In the *dnaK*-free genomes, the median number of CDS was 2,220, with the upper and lower quartiles of 1,698 and 3,164. In comparison, the median CDS number was 3,629 (2,715–4,549) in the genomes with a single copy of *dnaK*, and 6,832 (4,766–8,123) in the genomes with multiple copies of *dnaK*. The number of CDS in the genomes with no *dnaK* was significantly lower than that in the genomes with single *dnaK*, and the genomes with multiple copies of *dnaK* had the highest CDS number (*P* < 0.001). Similarly, in terms of multi-domain proteins, prokaryotes with multiple *dnaK* copies exhibited significantly higher proportions of proteins with multiple domains compared to those with a single copy of *dnaK*, which in turn was higher than the *dnaK*-free prokaryotes (Fig. 1D; Fig. S2; *P* < 0.001). These results strongly suggested that the occurrence and duplication of *dnaK* are in accordance with the increase of proteomic complexity, thus helping host cells to deal with more complex proteins (24).

We next computed the Ka/Ks ratio (rate of non-synonymous to synonymous substitutions) of the three functional regions of DnaK paralogs to determine their evolution pattern: Ka/Ks > 1 suggests positive selection in response to environmental pressure or functional changes, and Ka/Ks < 1 suggests purifying selection to maintain relatively stable structure and functions; higher or lower Ka/Ks values signify alteration extents under natural selection (25). In the prokaryotes with two DnaK homologs (those with more than two were not analyzed), with a few exceptions, the Ka/Ks value of the SBDα was almost always higher than that of the NBD or SBDβ (Data set S2). After removing the exceptions (collected in a separate table of Data set S2), the median Ka/Ks value was 0.22 (0.17–0.42) for the whole protein sequence, and specifically 0.20 (0.14–0.35) for the NBD, 0.24 (0.14–0.39) for the SBDβ, and 0.68 (0.43–1.28) for the SBDα (Fig. 1E). Thus, the NBD was the most conserved region, while the SBDα varied greatly. We noticed that there were some homologs with lower Ka/Ks for the SBD, suggesting their relatively conservative evolution after duplication. We examined the homologs with SBDβ or SBDα Ka/Ks values < 0.1, which were not phylogenetically clustered. Notably, in most of the DnaK homologs with lower Ka/Ks for the SBD, the sequence differences in the NBD were still smaller than that for the SBD.

The myxobacteria are a group of Gram-negative bacteria characterized by social behavior and a complex life cycle. They have been recently upgraded from the *Myxococcales* order in *Deltaproteobacteria* to the *Myxococcota* phylum based on 120 conserved single-copy marker genes as well as rRNA genes (26). In total, 93 DnaK homologs were found in the 48 completely sequenced genomes of myxobacteria; 43 strains encoded two copies, four strains (*Nannocystis exedens* DSM 71, *Corallococcus exiguous* NCCRE002, *Nannocystis pusilla* DSM 53165, and *Pajaroellobacter abortibovis* BTF920548A/99-0131) encoded a single DnaK, and one strain (*Vulgatibacter incomptus* DSM 27710) encoded three. Phylogenetic analysis revealed that myxobacteria DnaK proteins were categorized into two distinct clades, and the phylogenetic relationships of homologs within each clade were highly consistent with the phylogenomic tree of strains (Fig. S3). The missing DnaK in the four single DnaK-containing strains was the DnaK1 homolog of *M. xanthus* DK1622 (MxDnaK1), while the third DnaK in *V. incomptus* DSM 27710 was the highly similar to MxDnaK2 homolog. These results suggested that the duplication of DnaK in myxobacteria occurred in the early days of evolution, and the functional importance of the different DnaK homologs might vary in different myxobacterial taxa.

### Interactomes of MxDnaK1 and MxDnaK2 compared with that of EcDnaK

To explore the functional evolution of DnaK copies, we identified and compared the interactomes of the two copies of DnaK of *M. xanthus* DK1622, as well as compared them with that of *E. coli* DnaK. MXAN_3192 and MXAN_6671 both harbor PRK00290 domains and are respectively named MxDnaK1 and MxDnaK2 in this study. The MxDnaK1 and MxDnaK2 proteins share 58% and 62% amino acid identity with EcDnaK, respectively, and ~60% identity with each other. The NBD, SBDβ, and SBDα regions of the two *M. xanthus* homologs share identity of 64%, 70%, and 30%, respectively (Fig. S4), indicating that the SBDα region has the highest variation among the three regions of MxDnaK1 and MxDnaK2.

**Fig 2:**
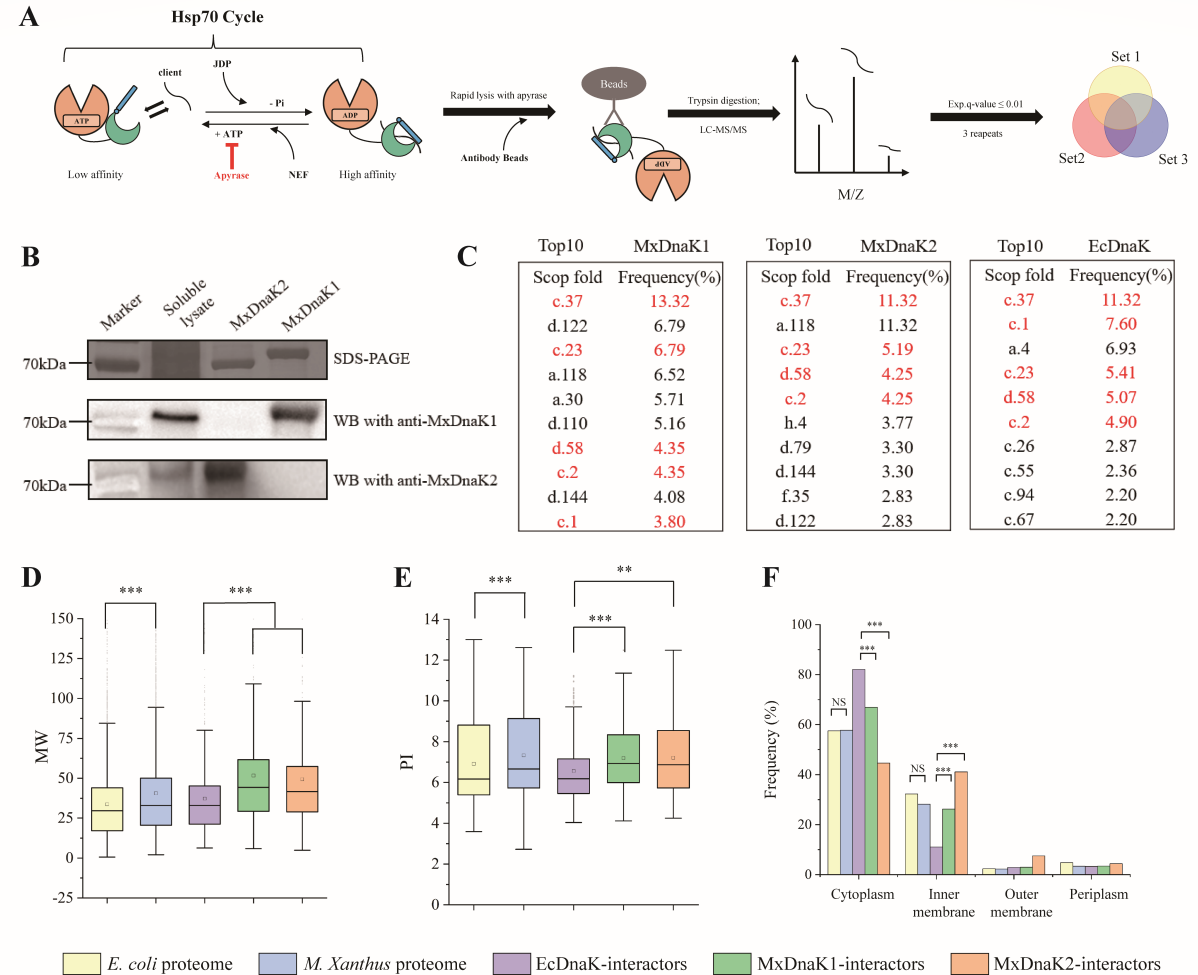
Identification and characterization of MxDnaK interactors. (**A**) A schematic diagram of Co-IP workflow for identifying MxDnaK interactors. LC-MS/MS: liquid chromatography-tandem mass spectrometry analysis (**B**) Western blot detection with antibodies raised against purified MxDnaK1 and MxDnaK2 fragments. The MxDnaK2 blotting band was weaker than that of MxDnaK1, consistent with their reported expression levels (11) (**C**) The top 10 SCOP folds of MxDnaK and EcDnaK interactors. (**D, E, and F**) Molecular weight (**D**), isoelectric point (**E**), and subcellular location (**F**) distribution of MxDnaK and EcDnaK interactors. The proteomes of *E. coli* and *M. xanthus* DK1622 served as controls. The *P*-value in (**D**) and (**E**) is based on the Mann-Whitney U-test, and the *P*-value in (**F**) is based on a χ^2^ test. NS, no significance, **P < 0.01 and ***P < 0.001.

We performed coimmunoprecipitation (Co-IP) experiments to identify the interacting proteins of MxDnaK1 and MxDnaK2 (Fig. 2A). The purified SBDα fragments of MxDnaK1 and MxDnaK2 were employed as antigens to generate specific antibodies. According to the western blotting results with the soluble lysate of *M. xanthus* DK1622 cells, as well as an MxDnaK1 mutant swapped with the MxDnaK2 SBDα region (YL2204, see below), the two antibodies specifically recognized MxDnaK1 and MxDnaK2 (Fig. 2B; Fig. S5 and S6). To stabilize the DnaK-substrate complexes, a high concentration of apyrase was added to the lysis buffer to deplete ATP, allowing the Hsp70 cycle to be stopped at the ADP-bound state (27). Negative controls for each MxDnaK (see Materials and Methods) were employed to identify proteins bound non-specifically to the beads or antibodies. Consequently, a total of 546 MxDnaK1 interactors and 375 MxDnaK2 interactors, including the JDP and NEF cochaperones, were identified by LC-MS/MS with *q*-value ≤0.01. Interestingly, the two interactomes were largely nonoverlapping (Data Set S3).

We compared the SCOP folds of the MxDnaK1 and MxDnaK2 interactors with the 674 published EcDnaK interactors (18). The MxDnaKs and EcDnaK all preferred α and β domain proteins, but had a low preference for proteins containing single secondary structure units (mainly α-helices or mainly β-sheets) (Fig. S7). For example, the proteins with SCOP fold c.37 (P-loop containing nucleoside triphosphate hydrolase) (18) are characterized by a complex α and β topology (28), and c.37 was the top protein structure interacting with DnaK in either *E. coli* or *M. xanthus* cells. Four and five of the top 10 preferred SCOP folds of EcDnaK (Fig. 2C) were also respectively detected in the MxDnaK1 and MxDnaK2 interactomes. Similarly, of the top 10 preferred SCOP folds of MxDnaK1 and MxDnaK2, seven were the same. A detailed comparison of the SCOP folds of the MxDnaK1 and MxDnaK2 interactors is provided in Data set S3. Thus, the DnaK interactors had a similar structure classification, not only between the MxDnaKs and EcDnaK, but also between the MxDnaK1 and MxDnaK2, probably due to the high conservation of DnaK proteins (29).

Notably, compared to that of EcDnaK, the interactors of MxDnaK1 and MxDnaK2 were both significantly shifted to larger sizes (Fig. 2D; *P* < 0.001) and included some very large proteins (Data set S3). For example, the proteins larger than 100 kDa occupied only ~2.7% of the EcDnaK interactors, but reached ~7.8% and ~7.2% of the MxDnaK1 and MxDnaK2 interactors, respectively. The size of the proteins interacting with MxDnaK1 and MxDnaK2 had no significant difference (*P* = 0.287). The size shift of the *M. xanthus* interactors was consistent with significantly larger molecular weights (MWs) of the *M. xanthus* proteome (Fig. 2D, *P* < 0.001) and proportion of large proteins (>100 kDa) in the *M. xanthus* and *E. coli* proteomes (4.2% and 1.9%, respectively). Similarly, the isoelectric point (pI) values of the *M. xanthus* DK1622 proteome and the MxDnaK interactors were both significantly higher than that of *E. coli* MG1655 proteome and the EcDnaK interactors (Fig. 2E).

Although the proteomes of *M. xanthus* and *E. coli* showed similar subcellular location distributions, the interactors of MxDnaKs and EcDnaK were distributed differently (Fig. 2F). Approximately 80% of the EcDnaK interactors were predicted to be cytosolic, while ~11% were inner membrane proteins and ~3% were located at the outer membrane. In contrast, the proportion of cytoplasmic proteins among the MxDnaK1 and MxDnaK2 interactors were ~65% and ~45%, respectively, but the proportion comprising membrane proteins were ~25% and ~40%, respectively. These results suggested that, compared to EcDnaK, MxDnaKs, and especially MxDnaK2, have evolved to interact to a greater extent with membrane proteins.

### Substrate composition of the MxDnaK1 and MxDnaK2 interactomes

**Fig 3:**
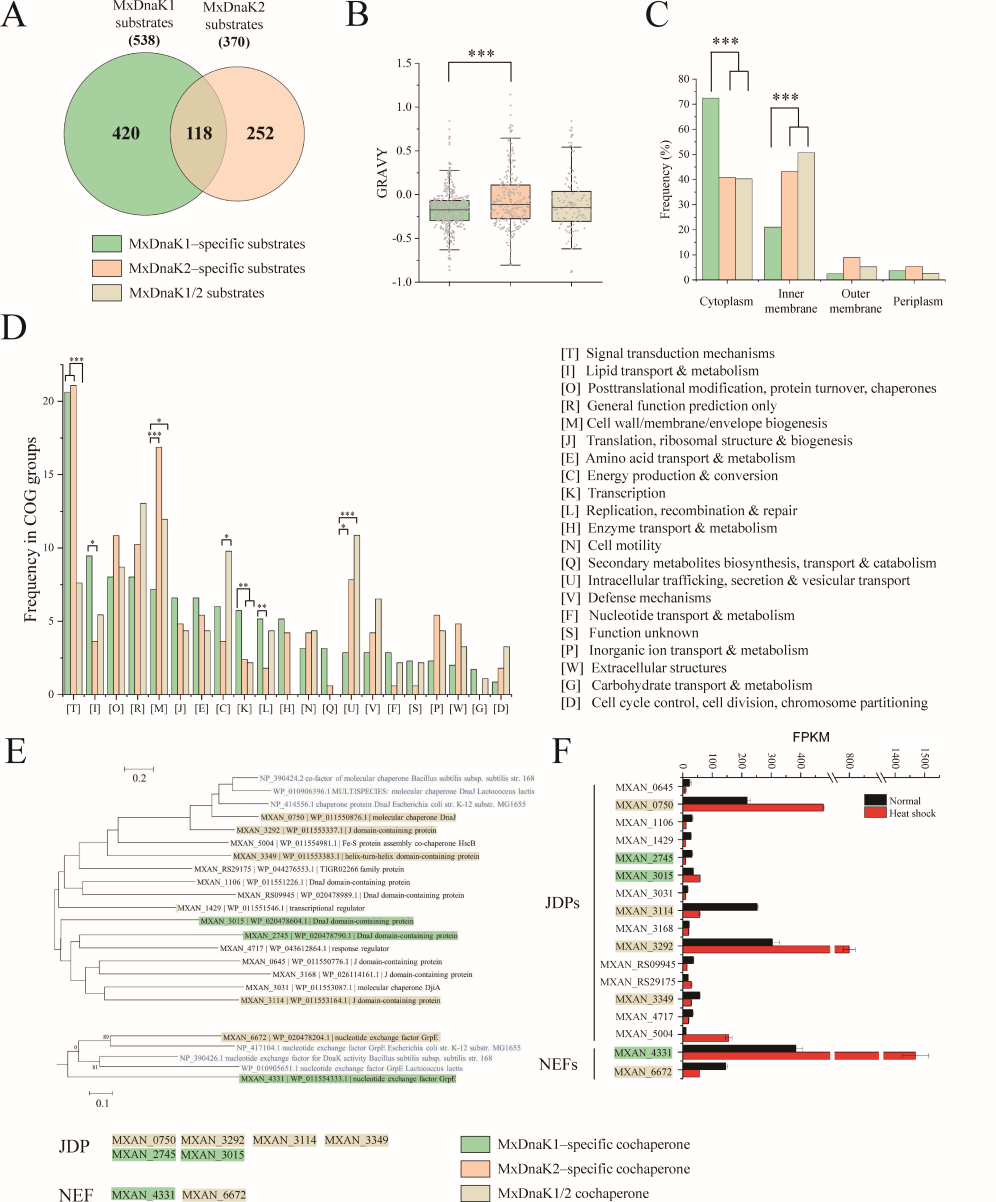
Analysis of MxDnaK1 and MxDnaK2 substrates. (**A**) Venn diagram of substrates of MxDnaK1 and MxDnaK2. (**B and C**) Average hydrophobicity (**B**) and subcellular localization (**C**) of MxDnaK1-specific, MxDnaK2-specific, and MxDnaK1/2 substrates. (**D**) Clusters of Orthologous Genes (COG) categories of MxDnaK substrates. The *P*-value in (**B**) is based on a χ^2^ test, in (**C**) and (**D**) is based on the Mann-Whitney U-test. *P < 0.05, **P < 0.01, and ***P < 0.001. (**E**) Phylogenetic tree of JDP and NEF homologs in DK1622. The typical JDPs and NEFs from *E. coli*, *B. subtilis,* and *L. lactis* (marked in blue) were employed for comparison. (**F**) Transcription levels of *dnaK* cochaperone genes in *M. xanthus* DK1622 under normal growth and heat shock conditions. FPKM represents the number of fragments per kilobase of transcript sequence per million base pairs sequenced.

Of the substrate clients interacting with MxDnaK1 and MxDnaK2 (not including the JPD and NEF cochaperones), 118 were bound to both MxDnaK1 and MxDnaK2 (MxDnaK1/2 substrates), while 420 and 252 were bound exclusively to MxDnaK1 and MxDnaK2 (MxDnaK1-specific substrates and MxDnaK2-specific substrates), respectively (Fig. 3A). These three substrate groups displayed similar MW and pI distributions (Fig. S8). However, the MxDnaK1- and MxDnaK2-specific substrates were significantly different with respect to their grand average of hydropathy (Gravy) (Fig. 3B, *P* < 0.001). Overall, the MxDnaK1-specific substrates were less hydrophobic than the MxDnaK2-specific substrates, which suggested that MxDnaK1 prefers to interact with thermally sensitive proteins compared to MxDnaK2 (30, 31). This result is consistent with the observation that MxDnaK1 is induced while MxDnaK2 is downregulated by heat shock (11).

Another interesting difference lies in the subcellular location of substrates. We previously determined that MxDnaK2 is involved in the oxidative stress response of *M. xanthus* DK1622 cells (11). As expected, MxDnaK2 preferred to interact with membrane proteins more than MxDnaK1 (*P* < 0.001), while the subcellular location distribution of MxDnaK1/2 substrates was similar to that of MxDnaK2-specific substrates (Fig. 3C). Comparatively, ~72% of the proteins that exclusively bound to MxDnaK1 (MxDnaK1-specific substrates) were cytosolic proteins, and ~20% were inner membrane proteins; this is similar to the distribution for EcDnaK substrates (18). The proteome of *M. xanthus* is much larger than that of *E. coli; M. xanthus* has two versions of DnaK and *E. coli* has one. Thus, we suggest that, to efficiently work with the increased diversity of proteins, MxDnaK2 has evolved more specifically to interact with membrane proteins, while MxDnaK1 persists more in the classical functions.

We predicted the substrate functions with the database of Clusters of Orthologous Genes (COGs), and the prediction ratios were 0.82 and 0.65 for the MxDnaK1 and MxDnaK2 substrates, respectively, suggesting that MxDnaK2 interacted with more function-unknown proteins than MxDnaK1. The MxDnaKs might play important roles in signal transduction in *M. xanthus*, as the COG with the highest proportion in both was signal transduction (COG class T in Fig. 3D). Comparatively, the MxDnaK1-specific substrates were preferentially involved in cellular processes of lipid transport and metabolism (COG class I) and transcription (COG class K), while the MxDnaK2-specific and MxDnaK1/2 substrates both preferred the membrane-associated proteins, including cell wall/membrane/envelope biogenesis (COG class M) and intracellular trafficking, secretion, and vesicular transport (COG class U). We also analyzed the substrates for Gene Ontology (GO), which showed that the MxDnaK1 clients were enriched in the Molecular Function category, such as proteins for adenyl nucleotide, ATP, and adenyl ribonucleotide binding, while the MxDnaK2 clients were enriched in the Biological Process group involving transport and location (Fig. S9). Thus, MxDnaK1 and MxDnaK2 have evolved to play divergent roles in different cellular processes, which seems to be consistent with the deletion phenotypes, i.e., the MxDnaK1 deletion is lethal, while the deletion of MxDnaK2 led to cellular sensitivity to oxidative stress and deficiency in social behaviors (11).

In *M. xanthus* DK1622, a total of 15 proteins are predicted to be JDPs (Data set S4), and none of them are neighbored by either of the genes encoding MxDnaK1 or MxDnaK2. Six JDPs were identified in the Co-IP assays: four (MXAN_0750, MXAN_3292, MXAN_3114, and MXAN_3349) bound to both MxDnaK1 and MxDnaK2, while two (MXAN_2745 and MXAN_3015) bound exclusively to MxDnaK1. The interactions between MxDnaK and each of the six JDPs were further confirmed by the split NanoBit luciferase (nLuc) assay (32) (Fig. S10). A phylogenetic analysis of the 15 JDPs based on their J domain sequences showed that two shared JDPs (MXAN_0750 and MXAN_3292) clustered in the same subbranch with the typical JDP proteins (class A JDPs) of *E. coli*, *Bacillus subtilis*, and *Lactococcus lactis* (Fig. 3E). The genes for these two JDPs exhibited high transcriptional levels and positive heat shock responses (Fig. 3F), which is consistent with that of MxDnaK1. However, the other two shared JDPs (MXAN_3114 and MXAN_3349) decreased their expression in response to heat shock, which is similar to that of MxDnaK2. While one of the main functions of JDPs is to interact with unfolded substrates and facilitate their delivery to Hsp70 (33), some substrates identified might bind to JDPs, rather than directly to Hsp70 (6, 8).

*M. xanthus* DK1622 possesses only two NEFs: MXAN_4331 and MXAN_6672, which are the homologs of *E. coli* GrpE with similarities of 32.69% and 34.43%, respectively. Of these two cochaperones, the transcription of *MXAN_4331* was highly induced by heat shock (Fig. 3F), which is consistent with that of the classical NEFs; whereas *MXAN_6672* was reduced by heat shock, which is similar to MxDnaK2. *MXAN_6672* lies upstream of *mxdnaK*2 (*MXAN_6671*), forming a bicistronic operon, as verified by real-time polymerase chain reaction (RT-PCR) (Fig. S11). As expected, MXAN_6672 was identified to interact with MxDnaK2. However, both NEFs were able to bind to MxDnaK1.

### Region inter-swapping between MxDnaKs and phenotypic effects

**Fig 4:**
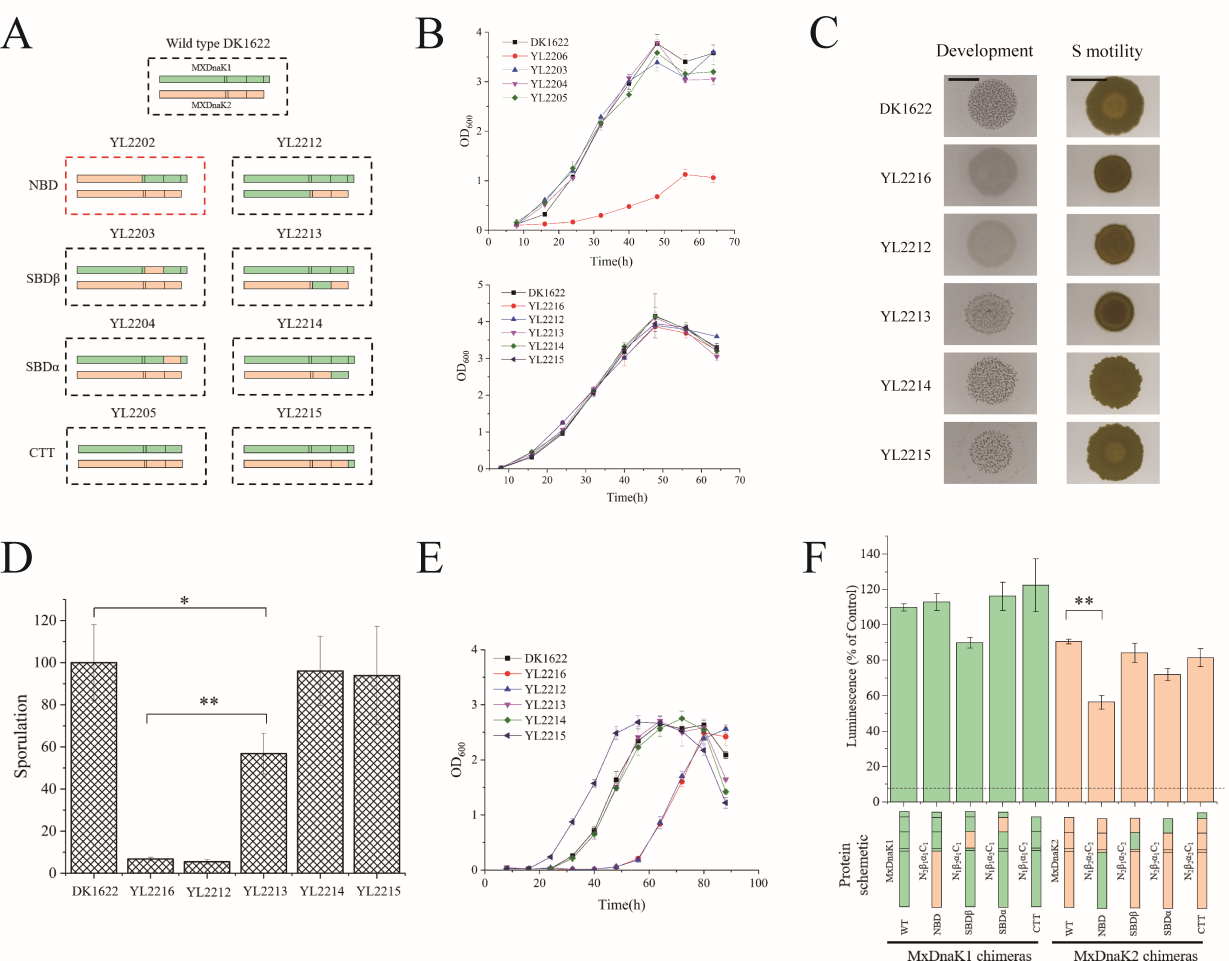
Region inter-swapping of MxDnaK1 and MxDnaK2 and their effects on cellular functions. (**A**) A schematic diagram of region inter-swapping. The swapping in YL2202 is lethal and the mutant is unavailable. (**B**) Growth curves of MxDnaK-swapping mutants compared to wild-type DK1622, MxDnaK1 (YL2206) and MxDnaK2 (YL2216) deletion mutants. (**C**) Fruiting body formation (scale bar, 4 mm) and social motility (on 0.3% agar; scale bar, 5 mm). (**D**) Starvation-induced sporulation ability comparison (percentage to that of DK1622). (**E**) Growth curves after 1.5-h treatment with H_2_O_2_. *In vitro* holdase activities of wild-type MxDnaKs and their chimeras. The chimeras were named according to N_x_β_x_α_x_C_x_, where X indicates the MxDnaK homolog a region is derived from. N, NBD; Β, SBDβ; α, SBDα; C, CTT. The dotted line represents the luminescence of the negative control, in which luciferase was heat treated with no chaperone. The *P*-value in (**D**) and (**F**) is based on two-tailed *t*-test. *P < 0.05, **P < 0.01.

To test the roles of different regions in determining functional specificity, we performed region inter-swapping between MxDnaK1 and MxDnaK2. The two *Myxococcus* DnaK proteins possess an almost identical linker sequence between their NBD and SBDβ (refer to Fig. S4), which likely permits stable conformation of the chimeras after domain exchange. We obtained *M. xanthus* mutants containing wild-type MxDnaK1 and a chimeric MxDnaK2 swapped with the NBD, SBDβ, SBDα, or CTT domain of MxDnaK1, or wild-type MxDnaK2 and a chimeric MxDnaK1 swapped with the SBDβ, SBDα, or CTT, but not the NBD domain of MxDnaK2 (Fig. 4A). In *M. xanthus* DK1622, MxDnaK1 is essential for cell survival, and the *in situ* deletion of the gene was obtained only after an insertion of a second copy of *mxdnaK1* in the genome at the *attB* site (11). To verify whether the NBD region is required for the essentiality of MxDnaK1, we performed region swapping of the *in situ* MxDnaK1 gene in the *att::mxdnaK1* mutant (a DK1622 mutant containing a second copy of *mxdnaK1*), and successfully obtained the MxDnaK1 mutant swapped with the MxDnaK2 NBD region. This result suggested that the NBD region of MxDnaK1 determines the essentiality of MxDnaK1 for cell survival, which was swapped only after an insertion of a second copy of the gene in the genome at the *attB* site (11). Notably, because the CTT domain is missing in MxDnaK2, we generated the corresponding mutants by directly deleting CTT from MxDnaK1 (YL2205) or fusing the MxDnaK1 CTT to the C-terminal of MxDnaK2 (YL2215).

We analyzed the growth abilities of these region-swapping mutants by comparing them with the wild-type strain, the *mxdnaK2* deletion mutant and a *mxdnaK1* depletion mutant as controls. The *mxdnaK1* depletion mutant (YL2206) was constructed by replacing the promoter of MxDnaK1 in DK1622 with the weak promoter J23112 (34), which resulted in only ~5% expression of MxDnaK1 (Fig. S12). As shown in Fig. 3B, YL2206 exhibited a significant growth defect, whereas all the inter-swapping mutants of MxDnaKs, as well as the *mxdnaK2* deletion mutant, had an almost identical growth curve to that of DK1622. These results indicated that high expression of MxDnaK1 is required for the normal growth of *M. xanthus* cells, while the inter-swapping mutants of MxDnaKs, if viable, have no effects on cellular growth.

We previously determined that the deletion of *mxdnaK*2 (strain YL2216) affected sporulation (~5% of wild type), fruiting body formation, S-motility, as well as the response to oxidative stress (11). Among the MxDnaK2 region-swapping mutants, the chimera with the NBD of MxDnaK1 (strain YL2212) showed almost identical phenotypes as the *mxdnaK*2 deletion mutant (Fig. 4C through E), which indicated that the NBD of MxDnaK2 is required for specific cellular functions of MxDnaK2. The strains carrying MxDnaK2 with the MxDnaK1 SBDβ (strain YL2213) showed similar defects in S-motility, sporulation ability (weaker than that of the NBD-swapping strain YL2212), but retained the ability to form fruiting bodies and oxidation resistance. These results indicated that the SBDβ swapping significantly, although not completely, limited the cellular functions of MxDnaK2. Comparatively, the exchange of SBDα or CTT had no obvious phenotypic impacts.

To determine whether the MxDnaK chimeras maintain chaperone functions, we assayed their *in vitro* holdase activity in protecting luciferase proteins from aggregation, which is often employed to measure the cochaperone-independent chaperone activities of DnaK (35). We expressed and purified the MxDnaK1 and MxDnaK2 proteins and their mutants. MxDnaK1 and MxDnaK2, despite having divergent cellular functions *in vivo*, exhibited similar *in vitro* holdase activities at various concentrations (Fig. S13). Interestingly, all the variants, including N_2_β_1_α_1_C_1_ (the MxDnaK1 chimera with the MxDnaK2 NBD), showed similar holdase activity as the wild-type protein except for N_1_β_2_α_2_C_2_ (the MxDnaK2 chimera with the MxDnaK1 NBD), which produced a relatively weak luminescence of heat-denatured luciferase (Fig. 4F). Thus, the region inter-swapping chimeras of MxDnaK1 or MxDnaK2 still retained at least the holdase activity of chaperones.

### Effects of NBD and SBDβ on the interactomes of MxDnaK1 and MxDnaK2

To explore possible contributions of the NBD and SBDβ to substrate recognition, we performed Co-IP with N_1_β_2_α_1_C_1_, N_1_β_2_α_2_C_2_ and N_2_β_1_α_2_C_2_ in YL2203, YL2212, and YL2213 to identify their substrates (Data set S3). Surprisingly, in comparison with the 538 and 370 substrates of wild-type MxDnaK1 and MxDnaK2, the substrate numbers of N_1_β_2_α_1_C_1_, N_1_β_2_α_2_C_2_ and N_2_β_1_α_2_C_2_ decreased to 150, 54, and 141, respectively. The results suggested that the swapping of NBD or SBDβ, especially the former, greatly decreased the interaction between substrates and DnaKs, although the cells with chimeric DnaKs exhibited no effect on growth.

**Fig 5:**
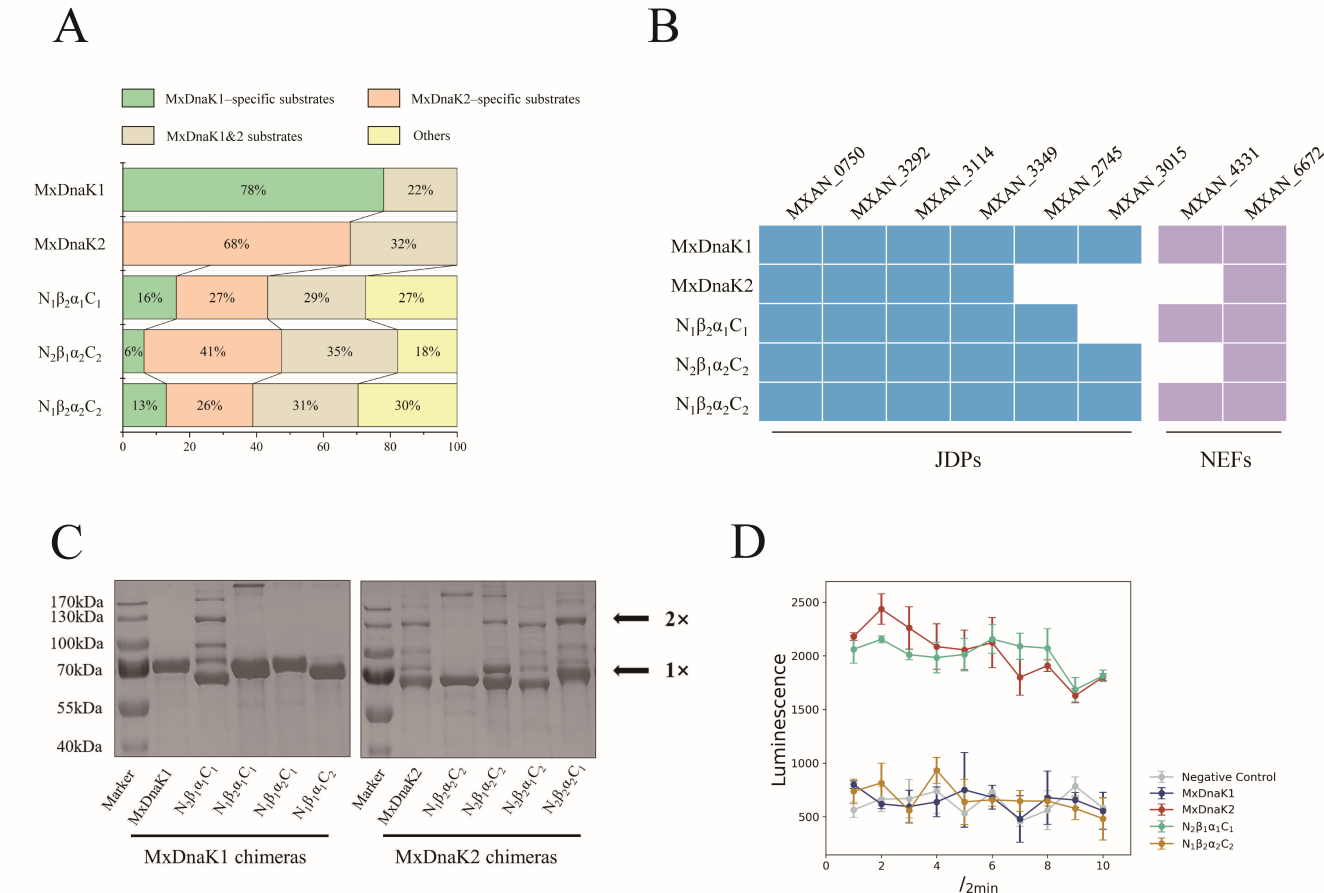
Effects of exchanging NBD and SBDβ. (**A**) Class distribution of chimera substrates compared to that of wild-type MxDnaK substrates. (**B**) Cochaperones distribution interacted by chimeras. (**C**) SDS-PAGE analysis of wild-type MxDnaKs and their chimeras, showing their oligomeric states. The positions of the monomer (1×) and dimer (2×) are indicated by arrows. The two distinct bands observed in the purified His6-MxDnaK2 lane, falling within the molecular weight range of 70–100 kDa, were identified as the interaction between His6-MxDnaK2 (65.3 kDa) and anti-FlhDC factor (WP_001300634.1, 27 kDa) as well as the 50S ribosomal protein L28 (WP_000091955.1, 9 kDa). (**D**) Split NanoLuc luciferase analysis of MxDnaKs and their NBD-swapping chimeras. Negative controls were performed with empty vectors.

Although the number decreased sharply, the substrates of these three chimeras overlapped extensively with that of the wild-type MxDnaKs: ~70%–82% substrates of the chimeras were in the substrate pools of MxDnaK1 or MxDnaK2 (Fig. 5A). The N_1_β_2_α_1_C_1_ substrates consisted of not only some of the original MxDnaK1 substrates but also some MxDnaK2-specific substrates. Similar results were obtained with N_1_β_2_α_2_C_2_ and N_2_β_1_α_2_C_2_ substrates. Notably, in contrast to the 22%–32% overlap of substrates between MxDnaK1 and MxDnaK2, 74 proteins overlapped in the interactomes of N1β_2_α_1_C_1_ and N2β_1_α_2_C_2_, accounting for ~50% of their respective total substrates (refer to Data set S3). Thus, the reduced number of substrates bound by the chimeras were still those essential components that are required for cellular growth, and those lost were mainly related to specific cellular functions like sporulation. In addition, some new substrates that were absent in the MxDnaK1 or MxDnaK2 interactomes were recognized by each of the three chimeras. These results not only validated the contribution of NBD and SBDβ in substrate recognition but also suggested that the swapping endowed the chimeras with some new functions.

In terms of cochaperones, the three chimeras were found to interact with all four MxDnaK1/2 JDPs (MXAN_0750, MXAN_3292, MXAN_3114 and MXAN_3349) but varied with the MxDnaK1/2-specific JDPs (Fig. 5B). For example, the exchange of SBDβ in N_1_β_2_α_1_C_1_ abrogated the binding to MXAN_3015, but still accepted MXAN_2745. In comparison, the exchange of SBDβ did not change the recognition of MxDnaK1 or MxDnaK2 to NEFs, while N_1_β_2_α_2_C_2_ bound to the same NEFs (both MXAN_4331 and MXAN_6672) as MxDnaK1. These results suggested that NBD plays an important role in NEF or JDP recognition, while SBDβ affected the recognition of JDPs only; the recognition of NEF and JDP did not affect each other.

In *E. coli*, DnaK proteins show a small amount of dimerization via disulfide bonds, which can be reduced by DTT (dithiothreitol) (35, 36). Interestingly, during expression and purification of the MxDnaK proteins, we found that MxDnaK2 formed an obvious dimer band, while MxDnaK1 presented only as a monomer band (Fig. S14 and Fig. 5C). We treated the MxDnaK2 proteins with 100 mM DTT, and the MxDnaK2 dimer was dissociated to the monomer form, with the band compositions were confirmed by LC-MS (Data set S5). We further tested the oligomeric states of MxDnaK1 and MxDnaK2 *in vivo* with nLuc assays (32). The MxDnaK proteins were fused with either the large (LgBiT) or the small (SmBiT) fragment of nLuc and co-expressed in an *E. coli* host. The formation of dimers was monitored by reconstituted nLuc activity. Consistent with the *in vitro* results, MxDnaK2-nLuc exhibited obvious nLuc activity, while MxDnaK1nLuc showed no difference from the negative control (Fig. 5D). The above results indicated that MxDnaK2, but not MxDnaK1, is prone to dimerize either *in vitro* or *in vivo*. Interestingly, the MxDnaK2 chimera with the NBD region of MxDnaK1 (N_1_β_2_α_2_C_2_) did not form the dimer band, while the MxDnaK1 chimera with the NBD region of MxDnaK2 (N_2_β_1_α_1_C_1_) formed an obvious dimer band (Fig. 5C). The *in vivo* nLuc assays also confirmed the dimeric state changes (Fig. 5D; Fig. S15). Thus, the NBD region is fully responsible for the difference of the dimeric state of MxDnaK1 and MxDnaK2, which is probably correlated with their substrate interacting activities, as well as the cochaperones.

## DISCUSSION

Hsp70 proteins are among the most ubiquitous chaperones and play important roles in maintaining proteostasis and resisting environmental stresses. To meet increased protein-refolding requirements, cells across all eukaryotes and ~40% prokaryotes encode multiple Hsp70 family members (8). Although some might be functionally redundant, Hsp70 family members generally exhibit a high degree of specialization. The differential expression, tissue-specific abundance, and subcellular localization of distinct Hsp70 isoforms are all suggested to contribute to diversification of the cellular functions of Hsp70s in eukaryotes (37, 38). The cellular proteomes of eukaryotes are more complex than those of prokaryotes, but the proteomic complexities in prokaryotes are also in hierarchy. We found that the presence of additional paralogs of the canonical bacterial Hsp70 (DnaK) is positively correlated with an increase in proteomic complexity, and bacteria that have evolved more and larger proteins for their complex life cycles, such as myxobacteria, are more likely to encode multiple DnaK proteins, helping cells maintain proteome integrity. Similarly, in eukaryotes, from *Dichelobacter nodosus* to *Homo sapiens*, the total number of proteins expands by approximately 90-fold, and the number of core-chaperone *hsp70* genes correspondingly increases by approximately 50-fold (24).

Due to high conservation, different Hsp70 paralogs are often regarded as functionally interchangeable (12, 39). However, functional specificity does exist between Hsp70 paralogs, consequently playing different cellular roles, e.g., the cytosolic SSA (stress-seventy subfamily A) and SSB proteins in yeast (17), DnaK, HscA, and HscC in *E. coli* (40), as well as MxDnaK1 and MxDnaK2 in *M. xanthus* (11). Although the two versions of DnaK in *M. xanthus* DK1622 both retain some shared clients, they are functionally different, which is consistent with their largely nonoverlapping substrates. We found that the substrates interacting with MxDnaK1 and MxDnaK2, or with MxDnaK1/2 and EcDnaK, have similar structures, molecular weights, and isoelectric points. MxDnaK1 prefers to bind to proteins without an extensive hydrophobic core, which are more unstable and aggregation prone in response to heat shock (31). In comparison, the MxDnaK1/2 substrates and MxDnaK2-specific substrates prefer membrane proteins, which is consistent with their COG or GO functional assignment. The subcellular location distribution of MxDnaK1-specific substrates was more like that of EcDnaK substrates. Furthermore, typical DnaK proteins are usually characterized by a high expression level and a positive heat shock response. For the two MxDnaKs in *M. xanthus*, MxDnaK1 is transcribed much higher than MxDnaK2 (approximately eightfold); the transcription of MxDnaK1 was increased, while that of MxDnaK2 was decreased by heat shock (11). Thus, MxDnaK1 exhibits similar transcriptional regulation and substrate preference as canonical DnaK (e.g., from *E. coli*), while MxDnaK2 appears to have evolved to support unique cellular processes in myxobacteria.

Region swapping was previously performed to explore the effects of Hsp70 homologs on phenotypes and specific substrates in yeast or human cells (41, 42). In this study, we performed region swapping between MxDnaK1 and MxDnaK2, and assayed their effects on cellular phenotypes. In addition, we identified the substrate pools of MxDnaK1 and MxDnaK2 chimeras to clarify the relationship between structural and functional divergence. Of the Hsp70 regions, the NBD and SBDβ are highly conserved, and are also important for binding substrates, whereas the SBDα and the CTT are not only rather variable, but also interchangeable with small effects on the chaperone functions. It has been previously hypothesized that the SBDβ plays a central role in the functional specificity of Hsp70 paralogs (43, 44). The importance of the NBD as a driver of functional specificity has also been experientially proven in human and yeast Hsp70 homologs (41, 45). In agreement with these findings, we found that the SBDβ was interchangeable between MxDnaK1 and MxDnaK2, while the exchange of the NBD led to similar phenotypes as the deletion of MxDnaK1 or MxDnaK2. Nevertheless, the NBD-swapped MxDnaK chimeras (N_1_β_2_α_2_C_2_ and N_2_β_1_α_1_C_1_) still retained their holdase function *in vitro*, and the region-swapping chimeras bound similar substrates, although the substrate numbers were greatly reduced. These results indicated that the exchange of NBD, as well as SBDβ, did not completely denature MxDnaK1 or MxDnaK2.

Perhaps one of the most surprising findings is the different oligomeric states of MxDnaK1 and MxDnaK2. The multitude of functions performed by Hsp70s, in addition to the cooperation with other cooperating protein-folding machineries like Hsp60s and Hsp90s, is specified by the cooperation with different Hsp70 family members, as well as the JDP and NEF cochaperones. The detected JDP and NEF cochaperones binding to the chaperones changed in the NBD-swapped chimeras. In contrast, only the JDP cochaperone changed in the SBDβ-swapping chimeras. These results are consistent with the interactions of these two domains, i.e., JDP binds at the interface between the NBD and the SBDβ, while NEF binds specifically to the NBD region (8). The corresponding changes in the substrate spectrums are thus suggested to be related not only to the shifts in cochaperone binding, but also to the changes in the domains. MxDnaK2, not MxDnaK1, is prone to form dimers both *in vitro* and *in vivo*. The Hsp70 oligomer has been confirmed to have a limited foldase activity, and to maintain holdase activity (46). In addition, mutation of the Hsp70 dimerization interface significantly decreased Hsp70-JDP affinity in both bacteria and mammals (36, 47). It is unsurprising that Hsp70 dimerization impedes its association with cochaperones and accordingly leads to defects in cochaperone-associated refolding activity (48). Similarly, our results demonstrated that exchange of the NBD not only reversed the oligomeric states of MxDnaK1 and MxDnaK2, but also exchanged their individual JDPs. JDPs play a crucial role in this process as they possess the ability to bind substrates and facilitate their targeted delivery. Hence, the oligomeric states, which consequently impact DnaK interactions with JDPs, also contribute to the disparities in substrate recognition between MxDnaK1 and MxDnaK2.

In this study, for the first time, we characterized the interactomes of bacteria Hsp70 paralogs, and determined the importance of NBD and SBDβ in their functional diversity based on phenotype and substrate identification. The reasons for the importance and cooperation of different regions are still unclear, of which our studies provide some important clues. The multitude of functions performed by Hsp70s are specified through multilayered networks of JDP and NEF cochaperones. Indeed, cochaperones have been reported to influence the functional diversity of Hsp70 in eukaryotes (33, 38). For example, the diverse preference of NEFs leads to opposing effects of HSPA1A and HSPA1L (Hsp70 homologs in *Homo sapiens*) on the fate of superoxide dismutase 1 mutants (42). We also found that the NBD and the SBDβ played important roles in JDP and NEF recognition by MxDnaK proteins. Thus, it will be interesting to further explore the relationships between the cochaperone recognition and the structural and functional divergence of Hsp70s.

## MATERIALS AND METHODS

### Distribution analysis of *dnaK*s in prokaryotes

The 16,083 representative prokaryotic genomes were obtained from the NCBI (National Center for Biotechnology Information) Reference Sequence Database. Conserved domain information of DnaKs and taxonomic information of prokaryotes were obtained from the Conserved domain database (49) and NCBI taxonomy database. All DnaK proteins were identified using RPS-BLAST (Reverse Position-Specific BLAST) based on the conserved domain PRK00290, and the retrieval condition was set to an *E*-value <0.01. The amino acid sequences were aligned using the protein sequence alignment program in MAFFT (50), and the *dnaK* gene sequences from representative prokaryotic genomes were retrieved from the NCBI database. The Ka/Ks values among the *dnaK* pairs were calculated using KaKs_Calculator 3.0 (51) with the MLWL (52). Phylogenetic trees were constructed and annotated by MEGA, CVtree3, and iTOL online.

### Strains, plasmids, and growth conditions

The strains, plasmids, and primers used in this study are listed in Tables S2 and S3. The *M. xanthus* strains were cultivated in CTT medium at 30°C. The *E. coli* strains were routinely grown in Luria-Bertani medium at 37°C. When required, 100 µg/mL ampicillin, 40 µg/mL kanamycin, 10 µg/mL tetracycline, or 20 µg/mL chloromycetin (final concentrations; Solarbio) was added to the solid or liquid medium.

### Hsp70 protein purification

Genomic DNA from *M. xanthus* DK1622 and region-swapping mutants served as a template for the PCR amplification of the *hsp70* genes. Subsequently, the genes were cloned into the pET28a vector and were fused with an N-terminal His tag gene. These constructs were then overexpressed in *E. coli* BL21 (DE3) cells upon induction with 0.1 mM isopropyl β-D-1-thiogalactopyranoside. After incubation at 16°C for 22 h, the BL21 cells were resuspended in resuspension buffer [25 mM Tris-HCl, 200 mM NaCl, 5% (vol/vol) glycerol; pH 8.0] and sonicated on an ice-water slurry. The cell lysate was centrifuged at 12,000 rpm for 30 min to remove the debris, and then the supernatant was incubated with Ni^2+^-NTA (GE Healthcare) at 4°C for 2 h. After incubation, the Ni Sepharose was washed with resuspension buffer supplemented with 20 mM imidazole to remove non-specific binding proteins, and the Hsp70 proteins were eluted with elution buffer [25 mM Tris-HCl, 200 mM NaCl, 5% (vol/vol) glycerol, 250 mM imidazole, pH 8.0]. If necessary, centrifugal concentrators (Millipore) were used to concentrate purified proteins. And thrombin was used to removed His_6_ tag.

### Western blot and antibodies

Western blot was performed as described previously with minor modifications (53). Briefly, proteins were loaded into 12% SDS-PAGE gels and then transferred onto activated PVDF membranes. The polyvinylidene fluoride membranes were incubated with anti-Hsp70 antibodies in TBST buffer (Tris-buffered saline with Tween 20) [20 mM Tris-HCl, 500 mM NaCl, 0.05% (vol/vol) Tween 20; pH 7.5]. Then, the membranes were washed six times with TBST buffer and blotted with horseradish peroxidase-conjugated goat anti-rabbit secondary antibodies (Sigma). After washing with TBST buffer again, visualization was performed with enhanced chemiluminescence detection reagents (GE Healthcare) and a ChemiDoc Imaging System (Bio-Rad) with Image Lab (Bio-Rad) software.

Anti-Hsp70 antibodies were prepared by ABclonal Biotechnology Co., Ltd, China.

### Isolation of Hsp70-interactor complexes

*M. xanthus* strains were grown for 24 h in CTT medium to the midlogarithmic phase (~1 OD_600nm_, optical density at 600 nanometers). Then, the cultures were cooled and harvested by centrifugation at 8,000 rpm for 5 min. The cell pellets were washed twice with PBS (phosphate-buffered saline) and suspended in lysis buffer [10 mM Tris-HCl, 0.1% (vol/vol) Triton X-100, 10 mM MgCl_2_, 12.5 U/mL benzonase, 1× EDTA-free protease inhibitor cocktail, 50 U/mL apyrase; pH 8.0) (18). Apyrase was added to remove the ATP in order to promote tight binding of substrate by DnaK. After sonication on ice, the debris was removed by centrifugation (12,000 rpm, 30 min), and the supernatant was used as the starting material for Co-IP. Co-IP was performed using the Protein A/G Matrix Immunoprecipitation Kit (Beaverbio, China). Briefly, the supernatant was incubated at 4°C overnight with anti-Hsp70 antibodies and beads. Then, the bead-antibody-Hsp70-interactor complexes were washed three times with IP Washing buffer (Beaverbio), and the Hsp70-interactor complexes were finally eluted from the beads with IP Elution buffer (Beaverbio). We performed the Co-IP experiments three times, and the proteins identified in at least two of three independent experiments were regarded as the Hsp70 interactors.

Eluates were treated with the FASP (filter-aided sample preparation) method with minor modifications. Briefly, the samples were washed two times with UA buffer (urea buffer, 8 M urea, 0.1 M Tris pH 8.5) and then incubated at 56°C for 1 h with 100 mM DTT in UA buffer to reduce disulfide bonds. After DTT treatment, samples were alkylated with 50 mM iodoacetamide by incubation for 30 min in the dark. Then, the samples were washed three times with UA buffer and two times with ammonium bicarbonate (ABC) buffer (50 mM NH_4_HCO_3_). After washing, trypsin (Sigma) was added at a final concentration of 0.5 µg/mL, and the samples were incubated at 37°C overnight. Peptides were eluted with ABC buffer and then desalted with C18 tips (Millipore) according to the manufacturer’s protocol. The final samples were lyophilized at room temperature and resuspended in ultrapure water for LC-MS analysis.

### Protein identification and bioinformatic analysis

Protein identification by LC-MS/MS was conducted on Orbitrap Fusion Lumos (Thermo Fisher) by Shandong University core facilities for life and environmental sciences. Raw files were analyzed by Proteome Discoverer software, which comes with the instrument. The interactors were identified with an Exp. *q*-value ≤0.01. We implemented negative controls for each MxDnaK in order to eliminate potential non-specific interactions with Protein A/G beads or antibodies. Specifically, we conducted a Co-IP experiment without the presence of antibodies to evaluate any non-specific binding to the Protein A/G beads. Additionally, the mxdnak2 deletion mutant (strain YL2216) and the MxDnaK1-swapping strain with the MxDnaK2 SBDα (strain YL2204) were utilized to investigate the non-specific binding to the antibodies of MxDnaK2 and MxDnaK1, respectively. As the SBDα of MxDnaK1 was employed as antigen to generate antibodies, YL2204 cannot be recognized by anti-MxDnaK1 (Fig. S4).

Protein fold assignment was derived from the Structural Classification of Proteins—extended 2.07 superfamily database (https://scop.berkeley.edu/) (28, 54). Sublocation of client proteins was predicted with pSORTb 3.0 (https://db.psort.org/) (55). Theoretical pIs and MWs of protein sequences were predicted with Expasy (https://web.expasy.org/compute_pi/) (56). Functional assignment of client proteins was performed using the COGs database (https://www.ncbi.nlm.nih.gov/research/cog) and GO database (http://geneontology.org/) (57, 58). The substrate features of EcDnaK were provided in a previous study (18). Proteome information of *E. coli* MG1655 and *M. xanthus* DK1622 was obtained from NCBI, and the protein features were predicted as mentioned above.

### RNA-seq experiment

The RNA-seq data of *M. xanthus* DK1622 were provided in a previous study (11). Briefly, *M. xanthus* strains were grown in CTT medium with shaking at 200 rpm at 30°C to the midlogarithmic phase (~1 OD_600_) as seed liquid. Then, the cells were treated with heat shock at 42°C for 1 h. Three replicates of cells (wild type and heat shock) were harvested from 24-h cultures, and RNA-seq was performed by Novogene Co., Ltd., China.

### Split nLuc oligomerization assay

NanoBit luciferase is split into two fragments: SmBiT and LgBiT (32). Either SmBiT or LgBiT fragment was fused to the C-terminus of a MxDnaK, and the MxDnaK- SmBiT and MxDnaK- LgBiT fragments were cloned into the pET28A and pACYC Duet-1 plasmids, respectively. Then, the pET28A-MxDnaK-SmBiT and pACYC-MxDnaK-LgBiT expression vectors were transformed into the *E. coli* BL21(DE3) cells simultaneously. The negative control contained the empty pET28A and pACYC vectors without the MxDnaK fragments. The luminescence was monitored using the Nano-Glo Live Cell Assay System kit (#N2011, Promega).

Additionally, pET28A-JDP-SmBiT and pACYC-MxDnaK-LgBiT expression vectors were used to validate the interaction between DnaKs and JDPs. And αSyn served as the positive control (59).

### RT-PCR analysis

*M. xanthus* DK1622 cells were collected from 24-h cultures, which were inoculated into CTT medium at a final concentration of OD_600_ 0.04. The cultures were harvested after 24 h, and the RNA was extracted immediately using a bacterial RNA extraction kit (TaKaRa). The purified RNA extracts were reverse-transcribed to cDNA. The cotranscription of *mxdnak2* and *MXAN_6672* was tested by PCR.

### Region swapping

Region swapping of *hsp70* genes in *M. xanthus* was performed using positive-negative KG cassettes (60). The upstream and downstream homologous arms and different domain sequences were cloned from the DK1622 genome and fused with pBJ113 to form region-swapping plasmids, which were then transferred via electroporation into *M. xanthus* DK1622 cells as previously described. The selected colonies were inoculated onto CTT agar plates supplemented with 1.5% galactose (Sigma) for a second round of screening. The region-swapping mutants were identified based on their galactose resistance and kanamycin sensitivity phenotypes, as well as by PCR and sequencing verification. The CTT domain is missing in MxDnaK2. Thus, exchanging the CTT of MxDnaK2 with that of MxDnaK1 was performed by directly inserting or deleting CTT in their respective regions.

### Developmental assays

The developmental assays were performed according to the method used in a previous study (61). Briefly, *M. xanthus* cells were harvested at midlogarithmic phase and resuspended in TPM buffer (10 mM Tris-HCl, 8 mM MgSO_4_, 1 mM K_2_HPO_4_- KH_2_PO_4_; pH 7.6) to a final cell concentration of 5 × 10^9^ cells/mL. Eight-microliter aliquots of concentrated cell suspension were spotted onto TPM agar. The colonies were incubated at 30°C and monitored every 24 h under a dissection microscope. Three replicates of fruiting bodies were harvested in 0.5 mL of TPM buffer and incubated at 50°C for 2 h to kill vegetative cells. After sonication, the spore suspensions were serially diluted and plated on CTT agar. After 5 days, the sporulation rate was calculated as the number of colonies. Assays were performed with three biological replicates.

### Swarm assays

The swarm assays were performed according to the method used in a previous study (62). Briefly, *M. xanthus* cultures were harvested at midlogarithmic phase, washed three times with TPM buffer (pH 7.6), and resuspended to a final concentration of 5 × 10^9^ cells/ml. Aliquots (2 µL) were dropped onto 0.3% CTT agar. After 72-h incubation, the swarming size of *M. xanthus* cells was monitored.

### Growth and oxidative resistance analysis

*M. xanthus* strains were grown in CTT medium with shaking at 200 rpm at 30°C to the midlogarithmic phase (~1 OD_600_) as seed liquid. Then, the cells were inoculated at a final cell concentration of 0.04 OD_600_ and grown in CTT medium for 64 h with shaking at 200 rpm. The OD_600_ value was read every 8 h.

To assay growth under oxidative damage stress, the seed liquid was treated with 1.5 mM hydrogen peroxide (H_2_O_2_) for 30 min before inoculation. The culture time was accordingly extended to 88 h.

### Luciferase assays

The luciferase holdase activity of Hsp70 proteins and mutants was performed as described with minor changes (35). Native firefly luciferase (Promega) was diluted in holdase buffer (50 mM HEPES, 300 mM KCl, 10 mM MgSO_4_, and 20 mM DTT, pH 7.5) at a concentration of 0.032 µM. After mixing with Hsp70 at a 1:1 ratio, the luciferase was heated to 39.5°C for 8 min. Fifty microliters of the reaction mixture was transferred to a 96-well, opaque assay plate, and then 50 µL of 5% (vol/vol) SteadyGlo reagent (Promega) in glycine buffer (50 mM glycine, 30 mM MgSO_4_, 10 mM ATP, 4 mM DTT, pH 7.8) was added to each well. Next, the luminescence at 560 nm was measured. For the experiment, the negative control contained all but Hsp70 proteins, and the 100% control contained native luciferase without heat shock.

### Statistical analysis

Significant differences were analyzed statistically based on paired *t*-tests (two-tailed), χ^2^ tests, or Mann-Whitney U-tests by SPSS or Scipy: *P < 0.05, **P < 0.01 and ***P < 0.001.

## SUPPLEMENTAL MATERIAL

Data Set S1 msystems.01154-23-s0001.xlsx Occurrence of DnaKs in representative genomes of prokaryotes.

Data Set S2 msystems.01154-23-s0002.xlsx Ka/Ks values of DnaK pairs.

Data Set S3 msystems.01154-23-s0003.xlsx Identified interactors and substrates of MxDnaKs.

Data Set S4 msystems.01154-23-s0004.xlsx J-domain proteins in *M. xanthus* DK1622.

Data Set S5 msystems.01154-23-s0005.xlsx LC/MS analysis of MxDnaK2 dimerization.

Supplemental material msystems.01154-23-s0006.pdf Figures S1-S15; Tables S1-S3.

ASM does not own the copyrights to Supplemental Material that may be linked to, or accessed through, an article. The authors have granted ASM a non-exclusive, world-wide license to publish the Supplemental Material files. Please contact the corresponding author directly for reuse.
