# Supplementary material for: DnaK duplication and specialization in bacteria correlates with increased proteome complexity
Source: mSystems. 2024 Mar 26;9(4):e01154-23. doi: 10.1128/msystems.01154-23 (PMC11019930; doi:10.1128/msystems.01154-23)
Supplement: Supplemental material — Figures S1-S15; Tables S1-S3. [file msystems.01154-23-s0006.pdf]

Supplemental Material

DnaK duplicates and regionally evolves for the increase of proteomic complexity in bacteria

Zhuo Pan[1], Li Zhuo[1, 2], Tian-yu Wan[1], Rui-yun Chen[1], Yue-zhong Li[1, *]

[1] *State Key Laboratory of Microbial Technology, Institute of Microbial Technology, Shandong University, Qingdao 266237, China*

[2] *Suzhou Research Institute, Shandong University, Suzhou 215123, China*

Content

Figure S1, S2, S3, S4, S5, S6, S7, S8, S9, S9, S10, S11, S12, S13, S14 and S15

Table S1， S2 and S3

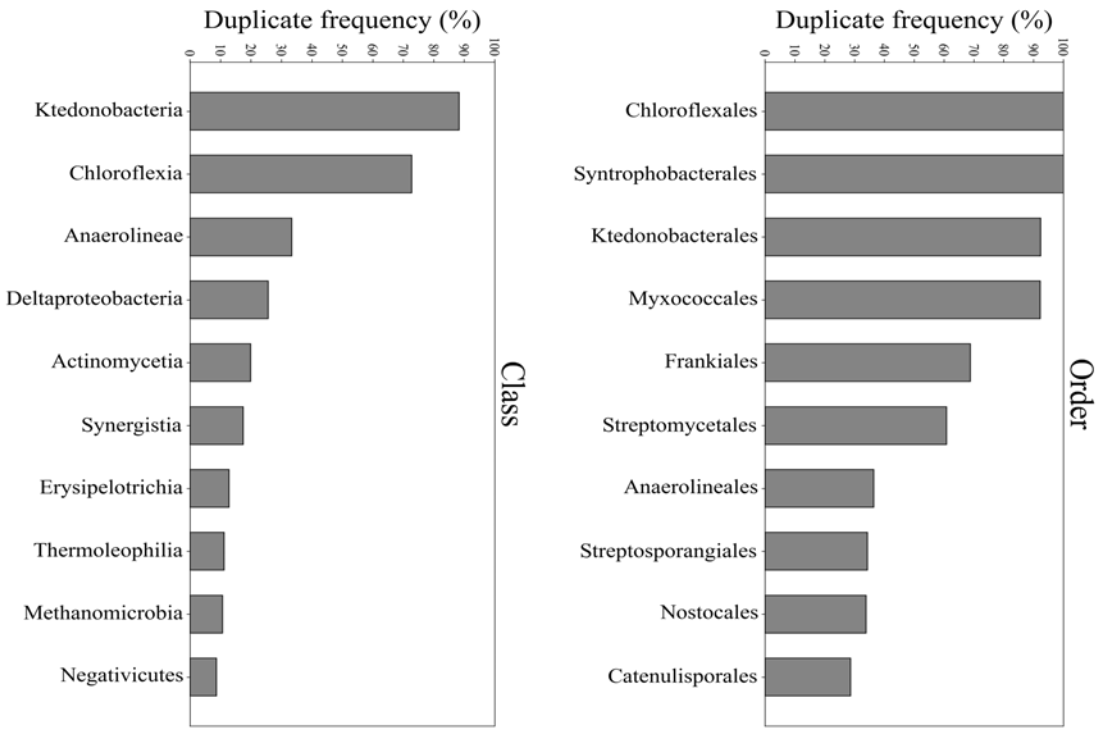

Figure S1. Occurrence ratio of DnaK paralogs in prokaryotes at the class and order level (top 10).

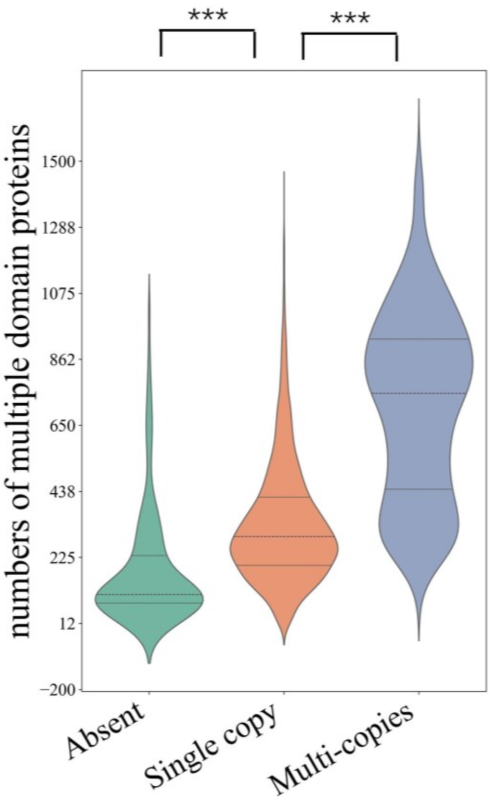

Figure S2. The multi-domain protein number of genomes with zero, single copy and multiple copies of *dnaK*.

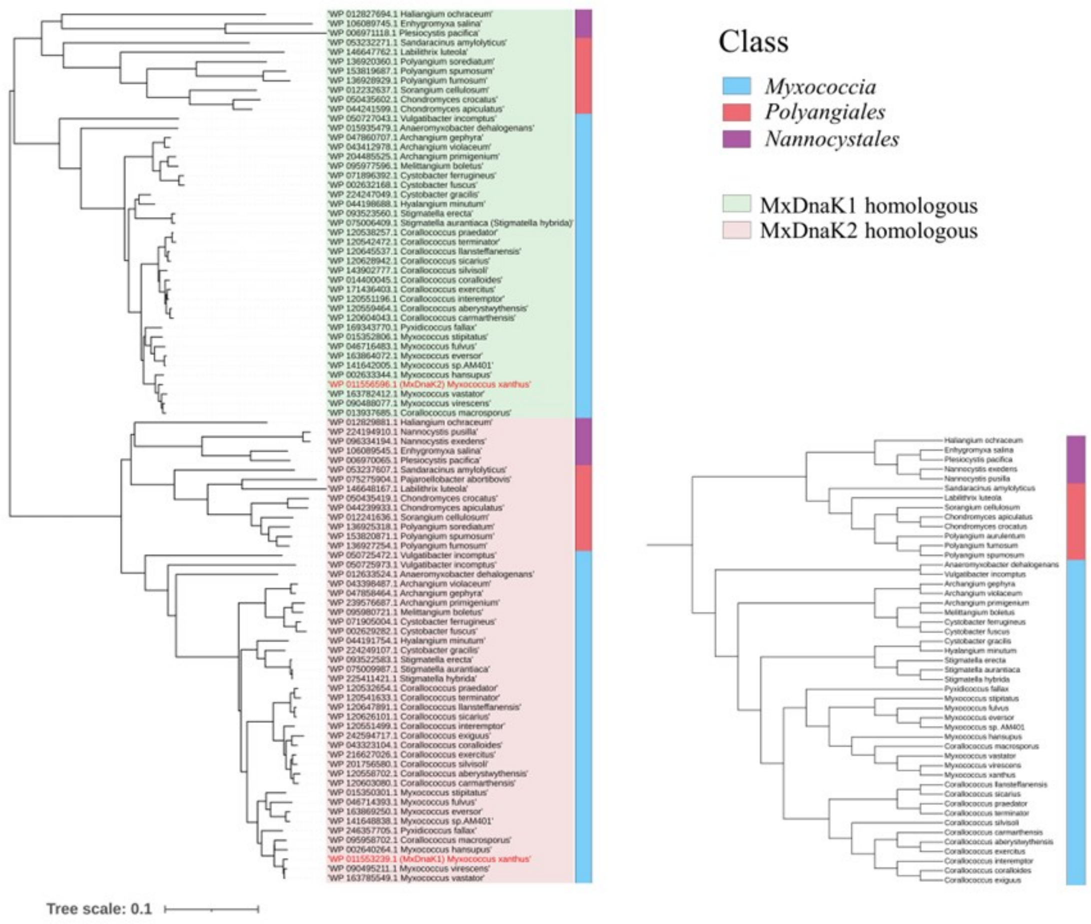

Figure S3. Phylogenetic analysis of myxobacterial DnaK proteins. Left: The phylogenetic tree of the 93 DnaK proteins from 48 myxobacteria. Right: The phylogenomic tree based on the 48 genome sequences.

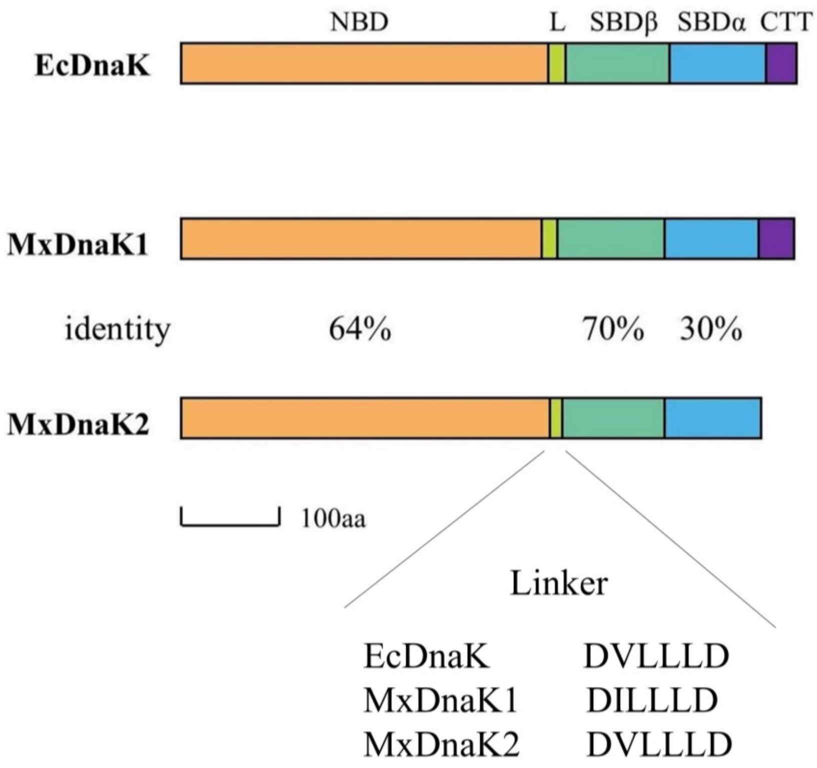

Figure S4. Domain organization and identity of MxDnaK1 and MxDnaK2. The EcDnaK (DnaK from *E. coli*) served as control. NBD: nucleotide binding domain; L: linker; SBDβ: substrate binding domain β; SBDα: substrate binding domain α; CTT: C terminal tail.

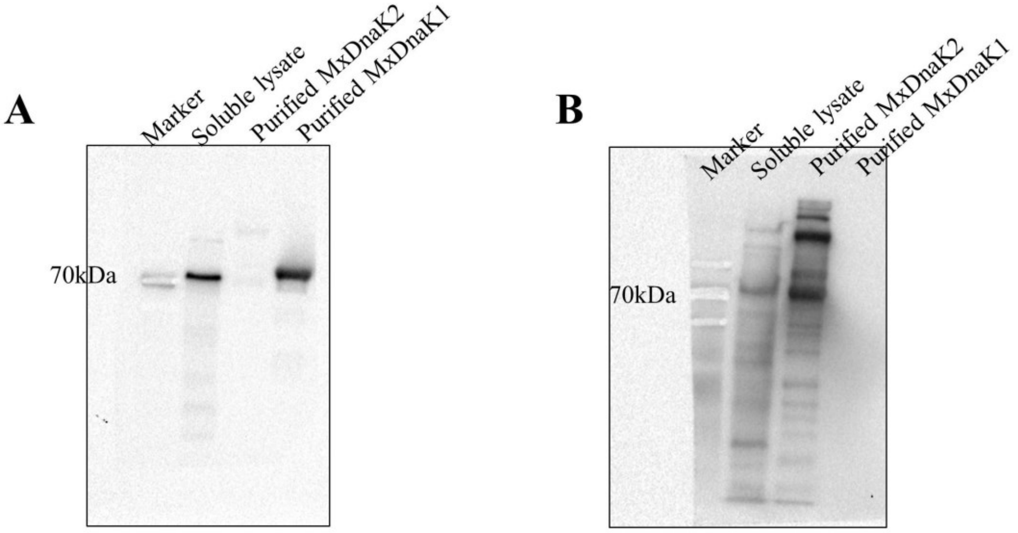

Figure S5. Western blot detection of the antibodies of MxDnaK1(A) and MxDnaK2(B) proteins. Soluble lysate represents the cell lysate of *M. xanthus* DK1622. both purified MxDnaK1 and MxDnaK2 proteins were engineered with an N-terminal His_6 tag.

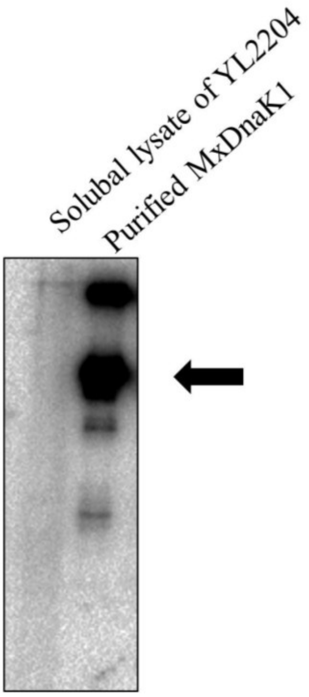

Figure S6. Western blot detection of the YL2204. YL2204 were employed to exclude the non-specifically bound proteins of MxDnaK1 antibodies.

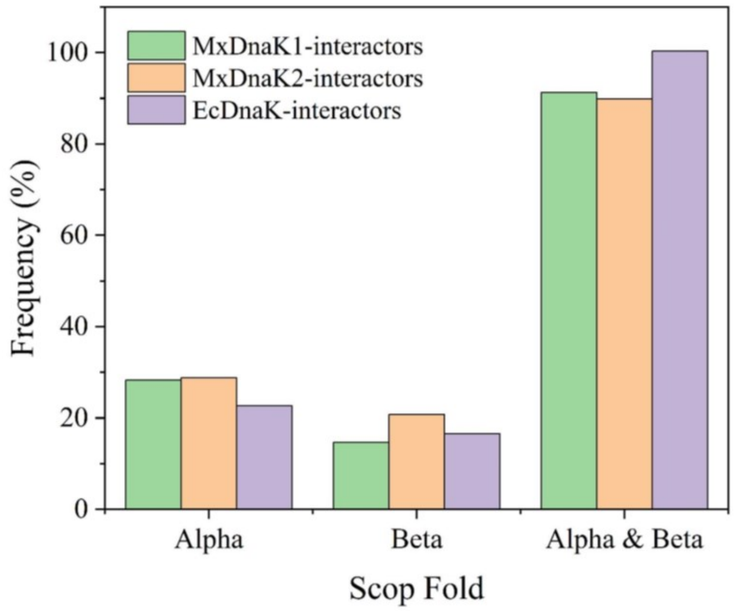

Figure S7. Secondary structure unit distribution for the MxDnaKs and EcDnaK (DnaK from *E. coli*) interactors. The protein fold assignment was derived from the SCOPe (Structural Classification of Proteins-extended) 2.07 superfamily database.

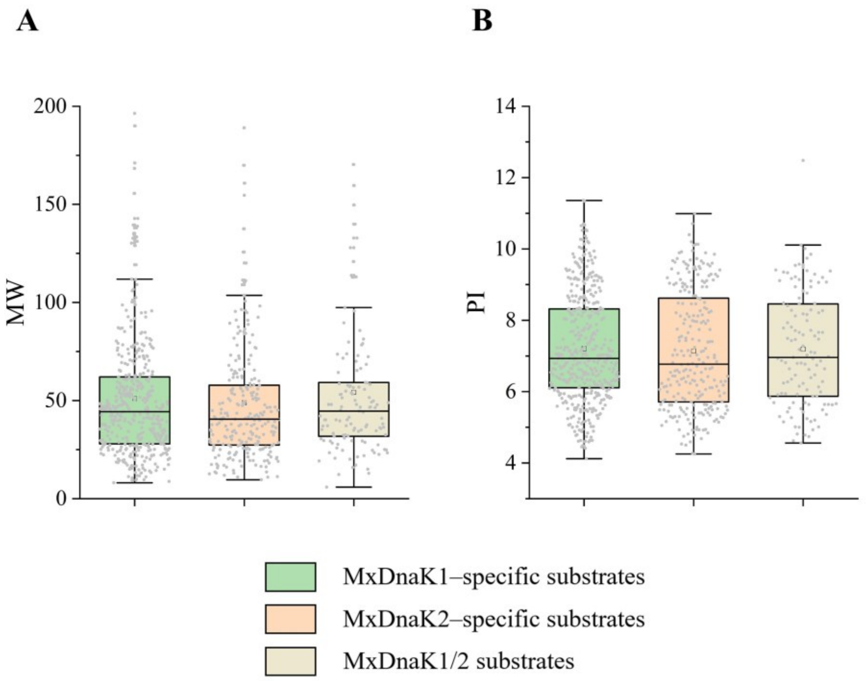

Figure S8. Molecular weight (A) and Isoelectric point (B) distribution of MxDnaK1-specific substrates, MxDnaK2-specific substrates and MxDnaK1/2 substrates.

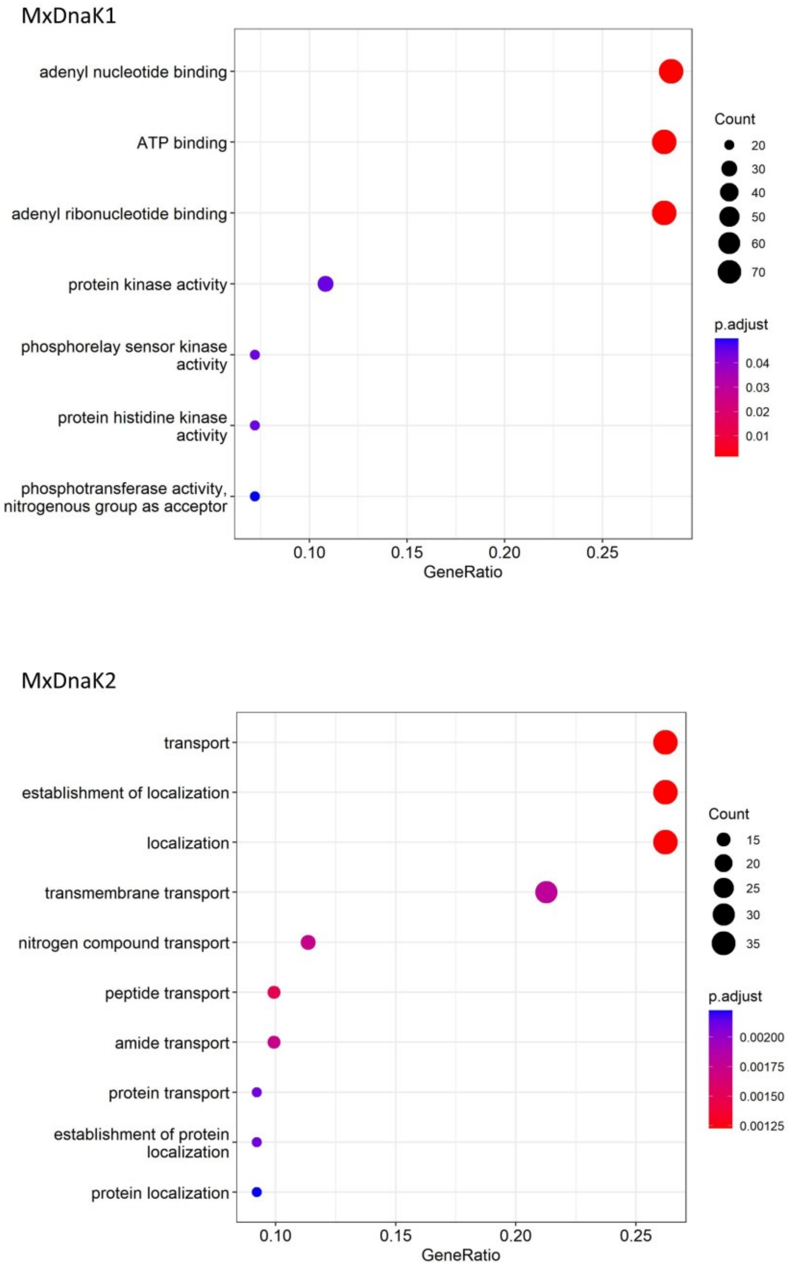

Figure S9. GO enrich analysis of MxDnaK1 and MxDnaK2 substrates.

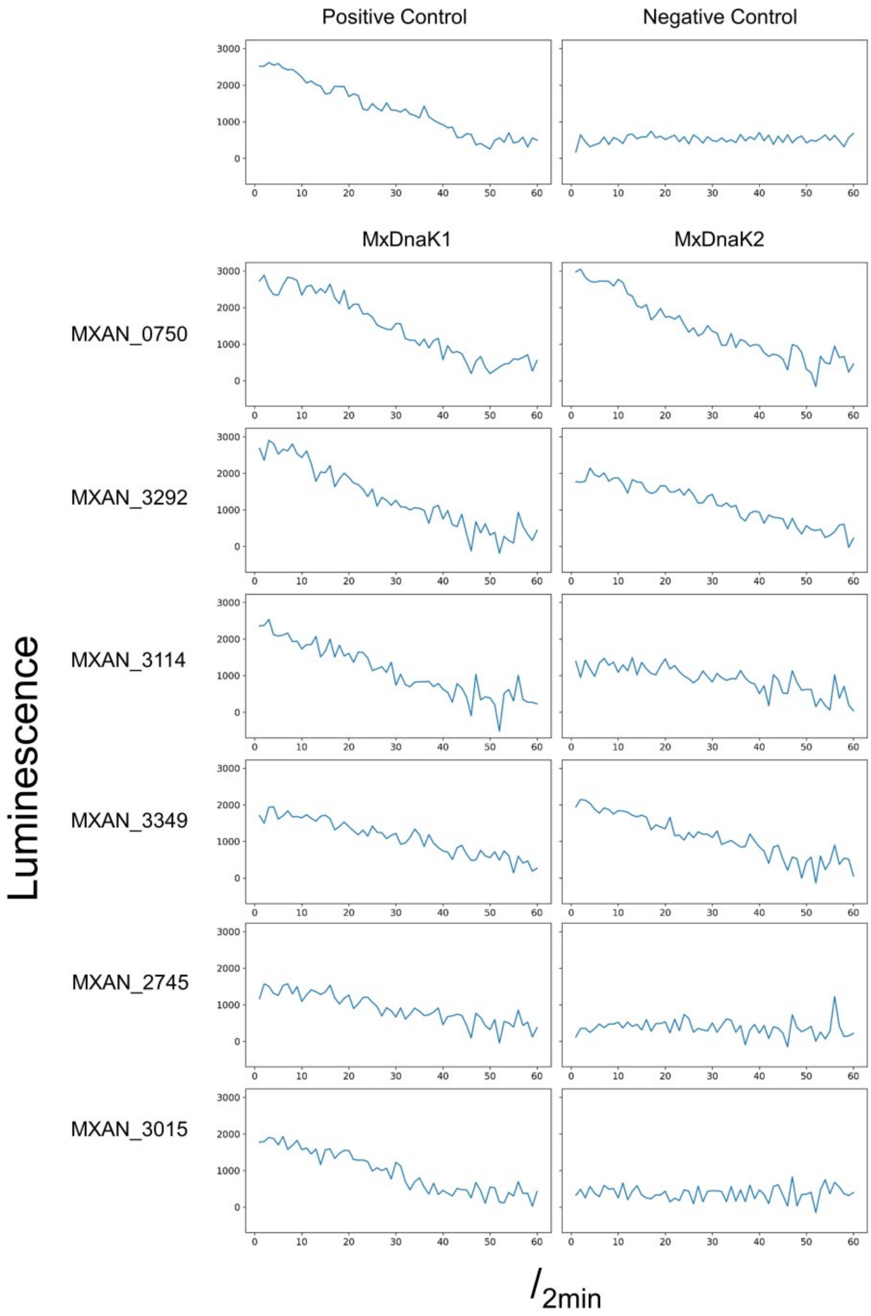

Figure S10. nLuc detection of the interaction between MxDnaKs and JDPs. Positive and Negative controls were performed with αSyn and empty vectors, separately.

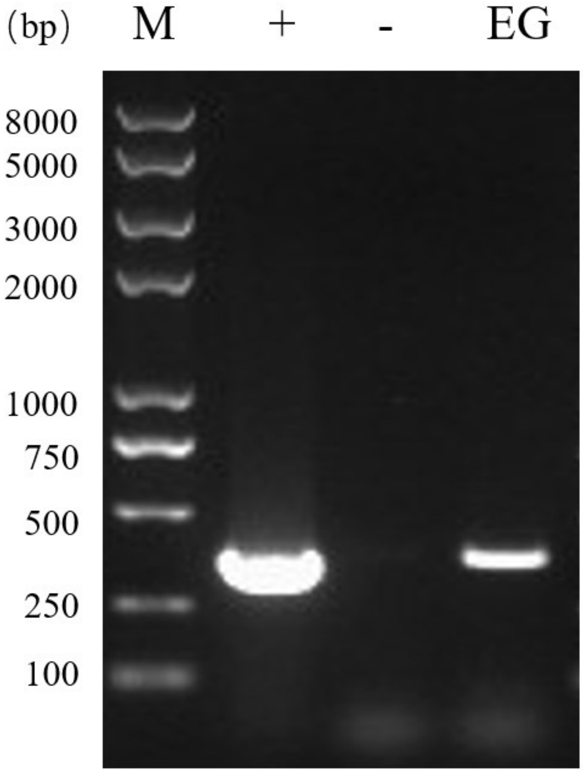

Figure S11. RT-PCR detection on the co-transcription of *mxdnaK2* (*MXAN_6671*) and *MXAN_6672*. M:marker; +: positive control using the total DNA extracted from DK1622 as the template; -:negative control in which no reverse transcriptase was added; EG: experimental group.

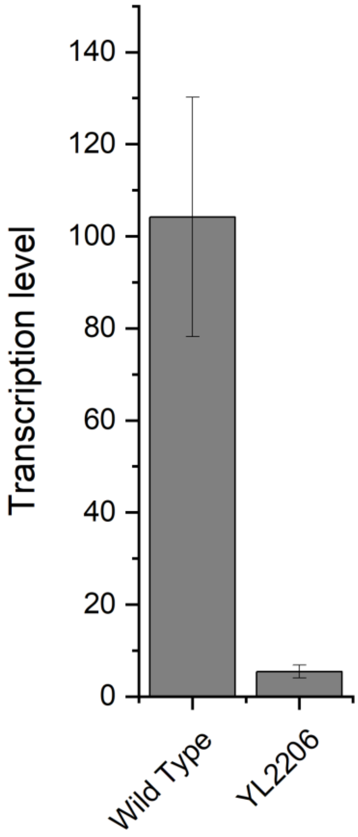

Figure S12. The transcription level of *mxdnaK1* in the *mxdnaK1* depletion mutant (YL2206). The YL2206 strain was generated by replacing the native promoter with the J23112 promoter.

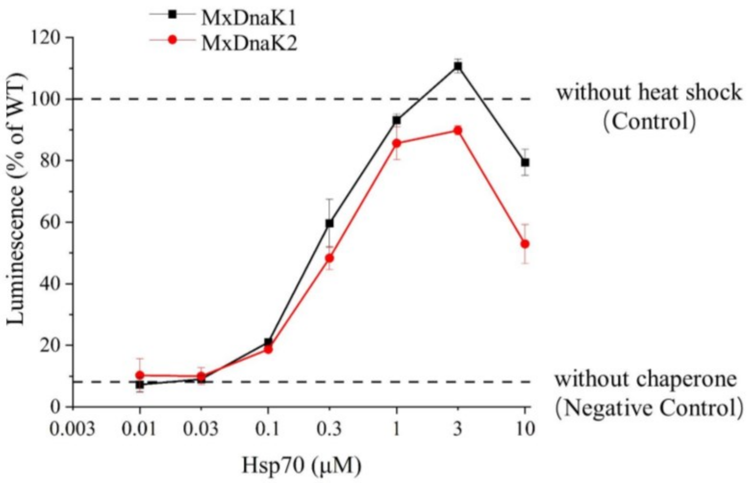

Figure S13. Holdase ability of various concentration of wild-type MxDnaK1 and MxDnaK2. The Y-axis (luminescence) was presented as the percent of control group in which the luciferase was not treated with heat shock (Control).

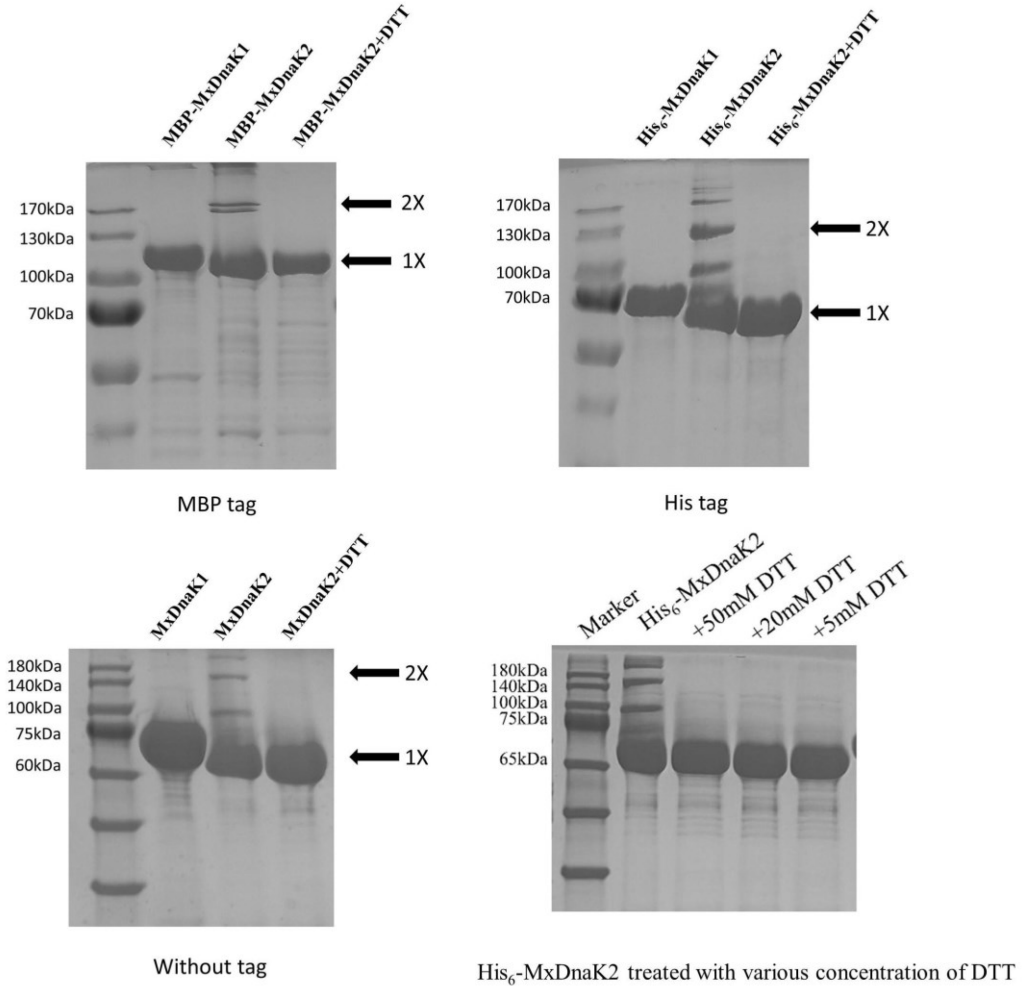

Figure S14. SDS-PAGE analysis of MxDnaK1 and MxDnaK2 with MBP or His_6 tag. The positions of the monomer (1X) and dimer (2X) are indicated by arrows. The His_6 and MBP tags were removed with thrombin to corroborate that they are not interfering with the dimerization.

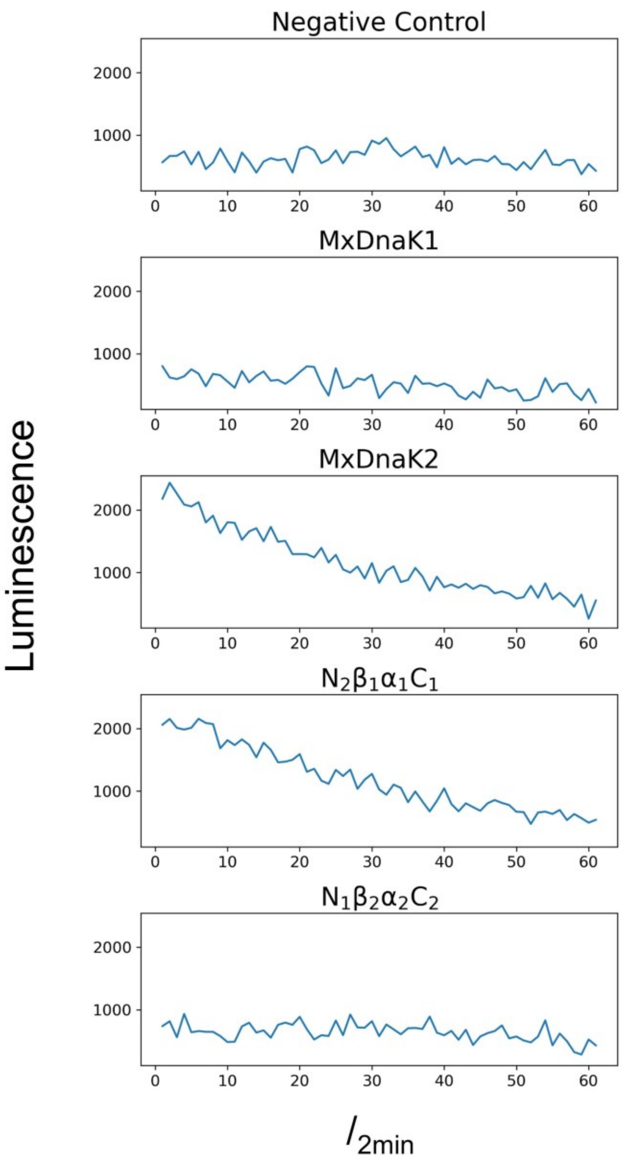

Figure S15. The complete 2-hours Split NanoLuc luciferase analysis of MxDnaKs and their NBD swapping chimeras. Negative controls were performed with empty vectors. According to the working principle of the nLuc assay, the amount of fluorescent substrate is limited. Therefore, even for proteins that form oligomers, the fluorescence value gradually decreases and reaches a plateau, similar to the negative control. This gradual decline in fluorescence is a significant indicator of protein interaction.

Table S1. The distribution of DnaKs in prokaryotes.

Phylum	Superkingdom	Genome number	DnaK number	Absent (%)	Single copy (%)	Multi-copies (%)
Chloroflexi	Bacteria	55	91	0	45.45	54.55
Armatimonadetes	Bacteria	4	6	0	50	50
Acidobacteria	Bacteria	38	51	0	68.42	31.58
Actinobacteria	Bacteria	3247	3982	0.95	79.67	19.37
Cyanobacteria	Bacteria	180	215	3.33	77.78	18.89
Synergistetes	Bacteria	23	27	0	82.61	17.39
Planctomycetes	Bacteria	115	119	0.87	95.65	3.48
Proteobacteria	Bacteria	6460	6634	0.68	96.11	3.2
Firmicutes	Bacteria	2845	2885	0.98	96.63	2.39
Verrucomicrobia	Bacteria	52	53	0	98.08	1.92
Euryarchaeota	Archaea	429	368	15.85	82.52	1.63
Spirochaetes	Bacteria	160	158	1.88	97.5	0.63
Bacteroidetes	Bacteria	1823	1810	1.1	98.52	0.38
Abditibacteriota	Bacteria	1	1	0	100	0
Fusobacteria	Bacteria	39	39	0	100	0
Ignavibacteriae	Bacteria	2	2	0	100	0
Gemmatimonadetes	Bacteria	5	5	0	100	0
Lentisphaerae	Bacteria	3	3	0	100	0
Kiritimatiellaeota	Bacteria	4	4	0	100	0
Rhodothermaeota	Bacteria	5	4	20	80	0
Nitrospinae	Bacteria	1	0	100	0	0
Nitrospirae	Bacteria	16	16	0	100	0
Tenericutes	Bacteria	191	166	13.09	86.91	0
Thaumarchaeota	Archaea	27	26	3.70	96.30	0
Thermodesulfobacteria	Bacteria	12	12	0	100	0
Thermotogae	Bacteria	42	42	0	100	0

candidate division NC10	Bacteria	1	1	0	100	0
Fibrobacteres	Bacteria	5	5	0	100	0
Deferribacteres	Bacteria	9	9	0	100	0
Elusimicrobia	Bacteria	3	3	0	100	0
Candidatus Korarchaeota	Archaea	1	0	100	0	0
Aquificae	Bacteria	22	14	36.36	63.64	0
Atribacterota	Bacteria	1	1	0	100	0
Balneolaeota	Bacteria	15	15	0	100	0
Caldiserica	Bacteria	1	1	0	100	0
Calditrichaeota	Bacteria	1	1	0	100	0
Candidatus Absconditabacteria	Bacteria	2	1	50	50	0
Candidatus Bipolaricaulota	Bacteria	1	1	0	100	0
Candidatus Cloacimonetes	Bacteria	2	2	0	100	0
Candidatus Cryosericota	Bacteria	1	1	0	100	0
Candidatus Kryptonia	Bacteria	4	4	0	100	0
Dictyoglomi	Bacteria	2	2	0	100	0
Candidatus Omnitrophica	Bacteria	1	1	0	100	0
Candidatus Saccharibacteria	Bacteria	6	4	33.33	66.67	0
Candidatus Thermoplasmatota	Archaea	14	13	7.14	92.86	0
Chlamydiae	Bacteria	30	28	6.67	93.33	0
Chlorobi	Bacteria	16	16	0	100	0
Chrysiogenetes	Bacteria	3	3	0	100	0
Coprothermobacterota	Bacteria	2	2	0	100	0

Crenarchaeota	Archaea	58	0	100	0	0
Deinococcus-Thermus	Bacteria	100	98	2	98	0
unclassified Bacteria	Bacteria	3	2	33.33	66.67	0
**Total**		**16083**	**16947**			

Table S2. Bacterial strains and plasmids used in this study.

Strain and plasmid	Genotype or description	Source or reference
Strains		
M. xanthus		
DK1622	Wild-type strain	D.Kaiser University of Standford
YL2201	DK1622::pSWU30-*MXAN_3192* Δ*MXAN_3192*	Previous study
YL2203	Exchange SBDβ domain of *MXAN_3192* with that of *MXAN_6671* in DK1622	This study
YL2204	Exchange SBDα domain of *MXAN_3192* with that of *MXAN_6671* in DK1622	This study
YL2205	Exchange CTT domain of *MXAN_3192* with that of *MXAN_6671* in DK1622	This study
YL2206	Exchange promoter domain of *MXAN_3192* with a weak promoter J23113 in DK1622	This study
YL2201	DK1622 Δ*MXAN_6671*	This study
att:: *mxdnaK1*	DK1622::pSWU30-*MXAN_3192* (*MXAN_3192* integrated at attB site with pilA promoter*)*	Previous study
YL2202'	Exchange NBD domain of *MXAN_3192* with that of *MXAN_6671* in *att*:: *MXAN_3192*	This study
YL2203'	Exchange SBDβ domain of *MXAN_3192* with that of *MXAN_6671* in *att*:: *MXAN_3192*	This study
YL2204'	Exchange SBDα domain of *MXAN_3192* with that of *MXAN_6671* in *att*:: *MXAN_3192*	This study

YL2205'	Exchange CTT domain of *MXAN_3192* with that of *MXAN_6671* in *att*:: *MXAN_3192*	This study
YL2212	Exchange SBDβ domain of *MXAN_6671* with that of *MXAN_3192* in DK1622	This study
YL2213	Exchange SBDβ domain of *MXAN_6671* with that of *MXAN_3192* in DK1622	This study
YL2214	Exchange SBDα domain of *MXAN_6671* with that of *MXAN_3192* in DK1622	This study
YL2215	Exchange CTT domain of *MXAN_6671* with that of *MXAN_3192* in DK1622	This study
YL2216	DK1622 Δ*MXAN_6671* (Δ*sglK*)	Previous study
E. coli		
BL21(DE3)	F^{-} *ompT* $hsdS_B(r_B^- m_B^-)$ *gal dcm* (DE3)	Tsingke company
DH5α	K-12,F-, Φ 80,-, Δ (argF-lac)169，lacZ58(M15), Δ phoA8，glnX44(AS),deoR481,rfbC1,gyrA96(NalR),recA1,endA1, thiE1,hsdR17	Tsingke company
H*mxdnak1*	pET28A- *mxdnak1* plasmid transformed into BL21(DE3)	This study
H*mxdnak2*	pET28A- *mxdnak2* plasmid transformed into BL21(DE3)	This study
H$mxdnak1^{NBD\text{-}2}$	pET28A- $mxdnak1^{NBD\text{-}2}$ plasmid transformed into BL21(DE3)	This study
H$mxdnak1^{SBD\beta\text{-}2}$	pET28A- $mxdnak1^{SBD\beta\text{-}2}$ plasmid transformed into BL21(DE3)	This study
H$mxdnak1^{SBD\alpha\text{-}2}$	pET28A- $mxdnak1^{SBD\alpha\text{-}2}$ plasmid transformed into BL21(DE3)	This study

H*mxdnak1* *CTT-2*	pET28A- *mxdnak1* $^{CTT-2}$ plasmid transformed into BL21(DE3)	This study
H*mxdnak2^{N}* *BD-1*	pET28A- *mxdnak2*$^{NBD-1}$plasmid transformed into BL21(DE3)	This study
H*mxdnak2* *SBDβ-1*	pET28A- *mxdnak2* $^{SBDβ-1}$ plasmid transformed into BL21(DE3)	This study
H*mxdnak2* *SBDα-1*	pET28A- *mxdnak2* $^{SBDα-1}$ plasmid transformed into BL21(DE3)	This study
H*mxdnak2* *CTT-1*	pET28A- *mxdnak2* $^{CTT-1}$ plasmid transformed into BL21(DE3)	This study
1-h2745	pACYC-Dute- *MXAN_2745* plasmid and pMAL-c5x-*MXAN_3192* co-transformed into BL21(DE3)	This study
1-h3015	pACYC-Dute - *MXAN_3015* plasmid and pMAL-c5x-*MXAN_3192* cot-ransformed into BL21(DE3)	This study
1-h0750	pACYC-Dute- *MXAN_0750* plasmid and pMAL-c5x-*MXAN_3192* co-transformed into BL21(DE3)	This study
1-h3292	pACYC-Dute - *MXAN_3292* plasmid and pMAL-c5x-*MXAN_3192* co-transformed into BL21(DE3)	This study
1-h3114	pACYC-Dute- *MXAN_3114* plasmid and pMAL-c5x-*MXAN_3192* co-transformed into BL21(DE3)	This study
1-h3349	pACYC-Dute - *MXAN_3349* plasmid and pMAL-c5x-*MXAN_3192* co-transformed into BL21(DE3)	This study
2-h0750	pACYC-Dute- *MXAN_0750* plasmid and pMAL-c5x-*MXAN_6671* co-transformed into BL21(DE3)	This study
2-h3292	pACYC-Dute - *MXAN_3292* plasmid and pMAL-c5x-*MXAN_6671*co-transformed into BL21(DE3)	This study
2-h3114	pACYC-Dute- *MXAN_3114* plasmid and pMAL-c5x-*MXAN_6671* co-transformed into BL21(DE3)	This study
2-h3349	pACYC-Dute - *MXAN_3349* plasmid and pMAL-c5x-*MXAN_6671* co-transformed into BL21(DE3)	This study

2-h1145	pACYC-Dute - *MXAN_1145* plasmid and pMAL-c5x-*MXAN_6671* co-transformed into BL21(DE3)	This study
mxdnak1^{NBD-2}-h0750	pACYC-Dute - *MXAN_0750* plasmid and pMAL-c5x-*mxdnak1*$^{NBD-2}$ co-transformed into BL21(DE3)	This study
mxdnak1^{NBD-2}-h3292	pACYC-Dute - *MXAN_3292* plasmid and pMAL-c5x-*mxdnak1*$^{NBD-2}$ co-transformed into BL21(DE3)	This study
mxdnak1^{NBD-2}-h1145	pACYC-Dute - *MXAN_1145* plasmid and pMAL-c5x-*mxdnak1*$^{NBD-2}$ co-transformed into BL21(DE3)	This study
mxdnak1^{NBD-2}-h3114	pACYC-Dute - *MXAN_3114* plasmid and pMAL-c5x-*mxdnak1*$^{NBD-2}$ co-transformed into BL21(DE3)	This study
mxdnak1^{NBD-2}-h3349	pACYC-Dute - *MXAN_3349* plasmid and pMAL-c5x-*mxdnak1*$^{NBD-2}$ co-transformed into BL21(DE3)	This study
mxdnak1^{NBD-2}-h2745	pACYC-Dute - *MXAN_2745* plasmid and pMAL-c5x-*mxdnak1*$^{NBD-2}$ co-transformed into BL21(DE3)	This study
mxdnak1^{NBD-2}-h3015	pACYC-Dute - *MXAN_3015* plasmid and pMAL-c5x-*mxdnak1*$^{NBD-2}$ co-transformed into BL21(DE3)	This study
Plasmids		
pBJ113	Gene replacement vector with KG cassette; Km^r	Laboratory collection
pET28A	Expression vector, Km^r,	Laboratory collection
pMAL-c5x	Expression vector, Amp^r,	Laboratory collection
pACYC-Dute	Expression vector, Km^r,	Laboratory collection
pET28A-*mxdnak1*	*mxdnak1* (*MXAN_3192*) insertion into pET28A	This study

pET28A-*mxdnak2*	*Mxdnak2* (*MXAN_6671*) insertion into pET28A	This study
pET28A-*mxdnak1^{NBD-2}*	*mxdnak1^{NBD-2}* insertion into pET28A	This study
pET28A-*mxdnak1 $^{SBDβ-2}$*	*mxdnak1 $^{SBDβ-2}$* insertion into pET28A	Laboratory collection
pET28A-*mxdnak1 $^{SBDα-2}$*	*mxdnak1 $^{SBDα-2}$* insertion into pET28A	This study
pET28A-*mxdnak1 $^{CTT-2}$*	*mxdnak1 $^{CTT-2}$* insertion into pET28A	This study
pET28A-*mxdnak2^{NBD-1}*	*mxdnak2^{NBD-1}* insertion into pET28A	This study
pET28A-*mxdnak2 $^{SBDβ-1}$*	*mxdnak2 $^{SBDβ-1}$* insertion into pET28A	This study
pET28A-*mxdnak2 $^{SBDα-1}$*	*mxdnak2 $^{SBDα-1}$* insertion into pET28A	This study
pET28A-*mxdnak2 $^{CTT-1}$*	*mxdnak2 $^{CTT-1}$* insertion into pET28A	This study
pACYC-Dute-*MXAN_0750*	*MXAN_0750* insertion into pACYC-Dute	Laboratory collection
pACYC-Dute-*MXAN_3292*	*MXAN_3292* insertion into pACYC-Dute	This study

pACYC-Dute - *MXAN_1145*	*MXAN_1145*insertion into pACYC-Dute	This study
pACYC-Dute - *MXAN_3114*	*MXAN_3114* insertion into pACYC-Dute	This study
pACYC-Dute - *MXAN_3349*	*MXAN_3349* insertion into pACYC-Dute	This study
pACYC-Dute - *MXAN_2745*	*MXAN_2745* insertion into pACYC-Dute	This study
pACYC-Dute - *MXAN_3015*	*MXAN_3015* insertion into pACYC-Dute	This study
pMAL-c5x-*mxdnak1*	*mxdnak1* (*MXAN_3192*) insertion into pMAL-c5x	This study
pMAL-c5x-*mxdnak2*	*Mxdnak2* (*MXAN_6671*) insertion into pMAL-c5x	This study
mxdnak1-NBD	Exchange NBD domain of *MXAN_3192* with that of *MXAN_6671* plasmid. The *MXAN_6671-NBD* and upstream and downstream of *MXAN_3192-NBD* were fused and insertion into pBJ113.	This study
mxdnak1-SBDβ	Exchange SBDβ domain of *MXAN_3192* with that of *MXAN_6671* plasmid.The *MXAN_6671*- SBDβand upstream and downstream of *MXAN_3192*- SBDβwere fused and insertion into pBJ113.	This study
mxdnak1-SBDα	Exchange SBDα domain of *MXAN_3192* with that of *MXAN_6671* plasmid. The *MXAN_6671*- SBDαand	This study

	upstream and downstream of *MXAN_3192*- SBDαwere fused and insertion into pBJ113.	
mxdnak1-CTT	Exchange CTT domain of *MXAN_3192* with that of *MXAN_6671* in plasmid. The *MXAN_6671*- CTT and upstream and downstream of *MXAN_3192*- CTT were fused and insertion into pBJ113.	This study
Mxdnak2-NBD	Exchange NBD domain of *MXAN_6671* with that of *MXAN_3192* plasmid. The *MXAN_3192-NBD* and upstream and downstream of *MXAN_6671-NBD* were fused and insertion into pBJ113.	This study
Mxdnak2-SBDβ	Exchange SBDβ domain of *MXAN_6671* with that of *MXAN_3192* plasmid.The *MXAN_3192* - SBDβand upstream and downstream of *MXAN_6671*- SBDβwere fused and insertion into pBJ113.	This study
Mxdnak2-SBDα	Exchange SBDα domain of *MXAN_6671* with that of *MXAN_3192* plasmid. The *MXAN_3192* - SBDαand upstream and downstream of *MXAN_6671*- SBDαwere fused and insertion into pBJ113.	This study
Mxdnak2-CTT	Exchange CTT domain of *MXAN_6671* with that of *MXAN_3192* in plasmid. The *MXAN_3192* - CTT and upstream and downstream of *MXAN_6671*- CTT were fused and insertion into pBJ113.	This study

Table S3. List of primers used in this study.

Primer	Sequence (5-3)*	Use
MXAN_3192-EXNBD-UF	TCGAGCTCGGTACCCAGATAGTGAGGCACC	Amplification of upstream homologous arm for exchange of NBD of MxDnaK1
MXAN_3192-EXNBD-UR	AATCACCTTGCCCATGGTTCCTATCCTCCG	
MXAN_3192-EXNBD -DF	TGTGCTCAAGGGCCTCTCCGGCGAGG	Amplification of downstream homologous arm for exchange of NBD of MxDnaK1
MXAN_3192-EXNBD -DR	CTCTAGAGGATCCCCAGTGAGAGGAGGAACGG	
MXAN_6671-NBD-F	CGGAGGATAGGAACCATGGGCAAGGTGATT	Amplification of NBD of MxDnaK2
MXAN_6671-NBD-R	CCTCGCCGGAGAGGCCCTTGAGCACA	
MXAN_3192-EX SBDβ-UF	TCGAGCTCGGTACCCTTCACGAAGGACGGG	Amplification of upstream homologous arm for exchange of SBDβ of MxDnaK1
MXAN_3192-EX SBDβ-UR	GACACCGAGCGACAGCGGCGTCAC	
MXAN_6671-SBDβ-F	GTGACGCCGCTGTCGCTCGGTGTC	Amplification of SBDβ of MxDnaK2
MXAN_6671-SBDβ-R	CTCCACCTCGTCCTTGGACAGGCCGGAGTT	
MXAN_3192-EXSBDβ -DF	AACTCCGGCCTGTCCAAGGACGAGGTGGAG	Amplification of upstream homologous arm for exchange of SBDβ of MxDnaK1
MXAN_3192-EXSBDβ -DR	CGACTCTAGAGGATCCCCCTTCAGATGCTCCAGGTGGA	
MXAN_3192-EX SBDα-UF	TCGAGCTCGGTACCCAAGAAGAAGGACGAG	Amplification of upstream homologous arm for exchange of SBD αof MxDnaK1
MXAN_3192-EX SBDα-UR	GATTTCCGCCTCCGCCAGACCGGA	
MXAN_6671-SBDα-F	TCCGGTCTGGCGGAGGCGGAAATC	Amplification of SBDα of MxDnaK2
MXAN_6671-SBDα-R	CGCGCCGCCCGTCGCCTGGCCCGT	

MXAN_3192-EXSBDα-DF	ACGGGCGGCGCGCCGGGTGCC	Amplification of upstream homologous arm for exchange of SBDα of MxDnaK1
MXAN_3192-EX SBDα-DR	TCGACTCTAGAGGATCCCCAAAACCCTTTTCCTCCAGG	
MXAN_3192-EX CTT-UF	TCGAGCTCGGTACCCACCAACGGTGACACG	Amplification of upstream homologous arm for exchange of CTT of MxDnaK1
MXAN_3192-EX CTT -UR	CGAATCAGCTCGCCTGGGCGCGGTACATCT	
MXAN_3192-EX CTT -DF	CCAGGCGAGCTGATTCGGAAGCGGTGAGCCG	Amplification of downstream homologous arm for exchange of CTT of MxDnaK1
MXAN_3192-EX CTT -DR	CTCTAGAGGATCCCCAGATGTCGTGCTGCC	
MXAN_6671-EXNBD-UF	CGAGCTCGGTACCCACAGGGACAGGTGG	Amplification of upstream homologous arm for exchange of NBD of MxDnaK2
MXAN_6671-EXNBD-UR	GCAGGCCGGCGTGGAGGTGAAGGACG	
MXAN_3192-NBD-F	CGTCCTTCACCTCCACGCCGGCCTGC	Amplification of NBD of MxDnaK1
MXAN_3192-NBD-R	GGGGGAGTCAGTAACCAACGTGGGCAAGATTATCGGGA	
MXAN_6671-EXNBD -DF	GATAATCTTGCCCACGTTGGTTACTGACTC	Amplification of downstream homologous arm for exchange of NBD of MxDnaK2
MXAN_6671-EXNBD -DR	CTCTAGAGGATCCCCAAGAAGACGATTGGC	
MXAN_6671-EXSBDβ-UF	CGAGCTCGGTACCCAGAAGAGCGTCCGG	Amplification of upstream homologous arm for exchange of SBDβ of MxDnaK2
MXAN_6671-EXSBDβ-UR	TCGTCCGGTCTGGCGGAGGCGGAAATCCAG	
MXAN_3192-SBDβ-F	CTGGATTTCCGCCTCCGCCAGACCGGACGA	Amplification SBDβ of MxDnaK1
MXAN_3192-SBDβ-R	GTGACGCCGCTGAGCCTGGGTGTG	
MXAN_6671-EXSBDβ -DF	CACACCCAGGCTCAGCGGCGTCAC	Amplification of downstream homologous arm for exchange of SBDβ of MxDnaK2
MXAN_6671-EXSBDβ -DR	ACTCTAGAGGATCCCCTTCCATGGTGGGCTT	

MXAN_6671-EX SBDα-UF	CGAGCTCGGTACCCAGAAGAGCGTCCGG	Amplification of upstream homologous arm for exchange of SBDαof MxDnaK2
MXAN_6671-EX SBDα-UR	GAGATGTACCGCGCCAGCTGAACGCTCGCA	
MXAN_3192-SBDα-F	TGCGAGCGTTCAGCTGGCGCGGTACATCTC	Amplification SBDαof MxDnaK1
MXAN_3192-SBDα-R	AACTCCGGCCTGTCCAAGGACGAGGTGGAG	
MXAN_6671-EX SBDα-DF	CTCCACCTCGTCCTTGGACAGGCCGGAGTT	Amplification of downstream homologous arm for exchange of SBDαof MxDnaK2
MXAN_6671-EX SBDα-DR	GACTCTAGAGGATCCCCTTCCATGGTGGGCTTCA	
MXAN_6671-EX CTT -UF	TCGAGCTCGGTACCCAATCCAACAACGGGC	Amplification of upstream homologous arm for exchange of CTT of MxDnaK2
MXAN_6671-EX CTT -UR	CGCCGCCCGTGCTCGCCTGG	
MXAN_3192-CTT -F	CCAGGCGAGCACGGGCGGCG	Amplification CTT of MxDnaK1
MXAN_3192-CTT -R	GCGATTGCGAGCGTCTAAGACTGGCGGA	
MXAN_6671-EX CTT -DF	TCCGCCAGTCTTAGACGCTCGCAATCGC	Amplification of downstream homologous arm for exchange of CTT of MxDnaK2
MXAN_6671-EX CTT -DR	CGACTCTAGAGGATCCCCAACTGCTGGACG	
MXAN_3192-28A-F	ATCCGGATCCGTGGGCAAGATTAT	PCR amplification of *mxdnaK*1 for ligation with pET28a
MXAN_3192-28A-R	GAGCTCGAATTCCTAAGACTGGCGGA	
MXAN_6671-28A-F	AAATGGGTCGCGGATCCATGGGCAAGGT	PCR amplification of *mxdnaK*2 for ligation with pET28a
MXAN_6671-28A-R	GACGGAGCTCGAATTCTCAGCTCGCCTGGCC	
MXAN_3192-pMAL-c5x -F	ATCGTCGACGGATCCGTGGGCAAGATTATC	PCR amplification of *mxdnaK*1 for ligation with pMAL-c5x
MXAN_3192-pMAL-c5x -R	ACCTGCAGGGAATTCCTAAGACTGGCGGAA	

MXAN_6671-pMAL-c5x -F	CGTCGACGGATCCATGGGCAAGGTGA	PCR amplification of *mxdnaK*2 for ligation with pMAL-c5x
MXAN_6671-pMAL-c5x -R	CCTGCAGGGAATTCTCAGCTCGCCTGGC	
MXAN_0750-pACYC-Dute-F	ctcggcgcgcATGTCAGCGGCGG	PCR amplification of *MXAN_0750* for ligation with pACYC-Dute
MXAN_0750-pACYC-Dute-R	cttgtcgacctgcagTCAGCCGAACAGCTC	
MXAN_1145-pACYC-Dute-F	gagctcggcgcgcATGTTCCCCACGC	PCR amplification of *MXAN_1145* for ligation with pACYC-Dute
MXAN_1145-pACYC-Dute-R	tgtcgacctgcagCTAGCGCCGCCGG	
MXAN_2745-pACYC-Dute-F	gagctcggcgcgcATGTCCCTCCCAG	PCR amplification of *MXAN_2745* for ligation with pACYC-Dute
MXAN_2745-pACYC-Dute-R	agcttgtcgacctgcagTCAGCGGATCTTGAACG	
MXAN_ 3015 -pACYC-Dute-F	tcgagctcggcgcgcGTGAGTGCGCCAAAC	PCR amplification of *MXAN_3015* for ligation with pACYC-Dute
MXAN_ 3015 -pACYC-Dute-R	caagcttgtcgacctgcagCTAGAACGTCCAGCGCAGC	
MXAN_ 3114 -pACYC-Dute-F	tcgagctcggcgcgcATGAACGCGGCGGca	PCR amplification of *MXAN_3114* for ligation with pACYC-Dute
MXAN_ 3114 -pACYC-Dute-R	cttgtcgacctgcagCTACCTGCTGCGCGC	
MXAN_ 3292 -pACYC-Dute-	agctcggcgcgcATGGCGGACGACTAC	PCR amplification of *MXAN_3292* for ligation with pACYC-Dute

F		
MXAN_ 3292 - pACYC-Dute-R	agcttgtcgacctgcagCTAGAGCGTCAGCTCGC	
MXAN_ 3349 - pACYC-Dute-F	cgagctcggcgcgcATGAAGCCCTTCGA	PCR amplification of *MXAN_3349* for ligation with pACYC-Dute
MXAN_ 3349 - pACYC-Dute-R	agcttgtcgacctgcagCTACTTCCGGCCTGACTT	
6671-6672Qf	TGAAGCCCACCATGGAAG	PCR to verify the co-translation of *MXAN_6671* and *MXAN_6672*
6671-6672QR	GCGCGGCTTCTACCTCAATGAA	
